# Coordination of two kinesin superfamily motor proteins, KIF3A and KIF13A, is essential for pericellular matrix degradation by membrane-type 1 matrix metalloproteinase (MT1-MMP) in cancer cells

**DOI:** 10.1016/j.matbio.2022.01.004

**Published:** 2022-03

**Authors:** Valentina Gifford, Anna Woskowicz, Noriko Ito, Stefan Balint, B. Christopher Lagerholm, Michael L. Dustin, Yoshifumi Itoh

**Affiliations:** Kennedy Institute of Rheumatology, University of Oxford, Oxford, UK

**Keywords:** MT1-MMP, Cancer invasion, KIF3A, KIF13A, KIF9

## Abstract

MT1-MMP plays a crucial role in promoting the cellular invasion of cancer cells by degrading the extracellular matrix to create a path for migration. During this process, its localization at the leading edge of migrating cells is critical, and it is achieved by targeted transport of MT1-MMP-containing vesicles along microtubules by kinesin superfamily motor proteins (KIFs). Here we identified three KIFs involved in MT1-MMP vesicle transport: KIF3A, KIF13A, and KIF9. Knockdown of KIF3A and KIF13A effectively inhibited MT1-MMP-dependent collagen degradation and invasion, while knockdown of KIF9 increased collagen degradation and invasion. Our data suggest that KIF3A/KIF13A dependent MT1-MMP vesicles transport takes over upon KIF9 knockdown. Live-cell imaging analyses have indicated that KIF3A and KIF13A coordinate to transport the same MT1-MMP-containing vesicles from the *trans*-Golgi to the endosomes, and KIF13A alone transports the vesicle from the endosome to the plasma membrane. Taken together, we have identified a unique interplay between three KIFs to regulate leading edge localization of MT1-MMP and MT1-MMP-dependent cancer cell invasion.

## Highlights

• MT1-MMP promotes cancer invasion by degrading barrier ECM at the leading edge, and its localization is carried out by direct vesicle transport of MT1-MMP containing vesicles along the microtubule.
• We identified KIF3A, KIF13A, and KIF9 as kinesins involved in MT1-MMP-containing vesicle trafficking in HT1080 cells.
• KIF3A and KIF13A transport MT1-MMP-containing vesicles from the *trans*-Golgi to the endosomes. KIF13A alone then transports the vesicles from endosomes to the plasma membrane for extracellular matrix degradation.

## Introduction

Invasion and metastasis are the life-threatening features of invasive cancer. Cancer cells achieve this by losing their cell-cell adhesion property, increasing their motility, and gaining extracellular matrix (ECM) degrading activity for invasion. It has been shown that one of the crucial ECM-degrading enzymes allowing cancer cell invasion and metastasis is membrane-type I matrix metalloproteinase, MT1-MMP [1], [2], [3], [4], [5], [6]. MT1-MMP is a type-I transmembrane proteinase that belongs to the matrix metalloproteinase (MMP) family. MT1-MMP degrades various ECM components on the cell surface, including fibrillar collagen [5,7,8]. MT1-MMP also activates proMMP-2 and proMMP-13 on the cell surface, expanding proteolytic repertoire [5]. ProMMP-2 activation is essential for epithelial cancer cell invasion and growth since activated MMP-2, but not MT1-MMP, can degrade type IV collagen, a major basement membrane component [5,9]. MT1-MMP also cleaves membrane proteins, including CD44 [10], ICAM-1 [11], LRP1 [12], syndecan 1 [13], ADAM9 [14], Dll1 [15], EphA2 [16,17], modifying cell adhesion property and cellular signalling. Thus, MT1-MMP is considered to be a microenvironment and cell function modifier.

Localization of MT1-MMP at the leading edge of migrating cells is crucial to promote cellular invasion [5,18,19]. Leading-edge structures include filopodia, lamellipodia, invadopodia, and podosomes, and MT1-MMP localizes to all of these structures [5,18,19]. MT1-MMP also localizes at focal adhesion (FA) [20,21]. It is thought that localization of MT1-MMP is achieved by direct transport of MT1-MMP-containing vesicles to these membrane structures [22], but the mechanism of vesicle transport of MT1-MMP is still poorly understood. Vesicle transport is carried out by motor proteins, including kinesin superfamily proteins (KIFs), which transport vesicles and macromolecules along microtubules [23,24]. There are 45 KIFs in humans that are classified into three groups, N-, M- and C-kinesins, according to the position of the microtubule-binding motor domain [23]. KIFs that transport vesicles toward the cell periphery or the (+) ends of microtubules belong to N-kinesins, which form the largest subgroup of 39 KIFs and can be further divided into 11 subgroups [23,24]. N-kinesins have a motor domain at their N-terminus, followed by a neck region, a coiled-coil region, and a C-terminal tail region [23,24]. It is thought that each KIF can selectively recognize certain cargos through the specific interaction with adaptor molecules, membrane proteins, or Rab GTPases through their C-terminal tail region. So far, KIF5B and KIF3A/B have been reported to transport MT1-MMP vesicles in macrophages [22]. KIF5B and KIF3A have been reported to play a role in invadopodia localization of MT1-MMP in MDA-MB231 cells [25,26]. However, the roles of KIFs in MT1-MMP vesicles trafficking in different cells are not understood.

This study aimed to identify KIFs responsible for MT1-MMP localization at the leading edge of invasive human fibrosarcoma, HT-1080. We identified three KIFs (KIF3A, KIF13A, and KIF9) directly involved in MT1-MMP vesicle trafficking by screening with siRNAs. We report here the coordination of KIF3A and KIF13A to transport MT1-MMP vesicles and the potential competitive role of KIF9 against KIF3A and KIF13A.

## Results

### Knockdown of KIF13A, KIF3A, KIF9, and KIF1C alters MT1-MMP-mediated cell functions on the cell surface

To identify KIFs responsible for MT1-MMP vesicle transport, we initially narrowed down the candidate KIFs. There are 45 KIFs in humans, and we excluded C- and M-kinesins since they do not traffic to the (+) ends of microtubules. We further excluded KIFs reported to be exclusively expressed in neurons or involved in cell division as they are unlikely to control MT1-MMP vesicle trafficking (Supplementary Table S1). This exercise leaves 17 KIFs, including splicing variants, as candidates for screening. We chose HT-1080 cells to investigate MT1-MMP vesicle trafficking since the cells are well characterized for MT1-MMP-dependent cellular invasiveness and pericellular matrix degradation. To screen KIFs necessary for MT1-MMP trafficking, we set up a gelatin and collagen film degradation assay with HT-1080 cells. As shown in Supplementary Fig. S1a–d, gelatin and collagen film degradation were abolished by a broad-spectrum MMP inhibitor GM6001 (10 μM) and by a specific biologic MT1-MMP inhibitor DX-2400 (200 nM) [27], but not by TIMP-1 (200 nM), which does not inhibit MT1-MMP but inhibits all soluble and GPI-anchored MMPs. The data suggested that both gelatin and collagen film degradation activities solely depend on endogenous MT1-MMP in HT1080 cells. We confirmed that HT-1080 cells expressed all 17 KIFs (Supplementary Fig S2a), each KIF gene was silenced by siRNA, and cells were subjected to gelatin film degradation assay (Fig. S2b, c). Among the 17 KIFs, knockdown of KIF1C, KIF3A, and KIF13A notably decreased gelatin film degradation, while knockdown of KIF9 instead increased gelatin film degradation (Supplementary Fig. S2b). Therefore, we investigated KIF1C, KIF3A, KIF13A, and KIF9 further.

**Fig. 1:**
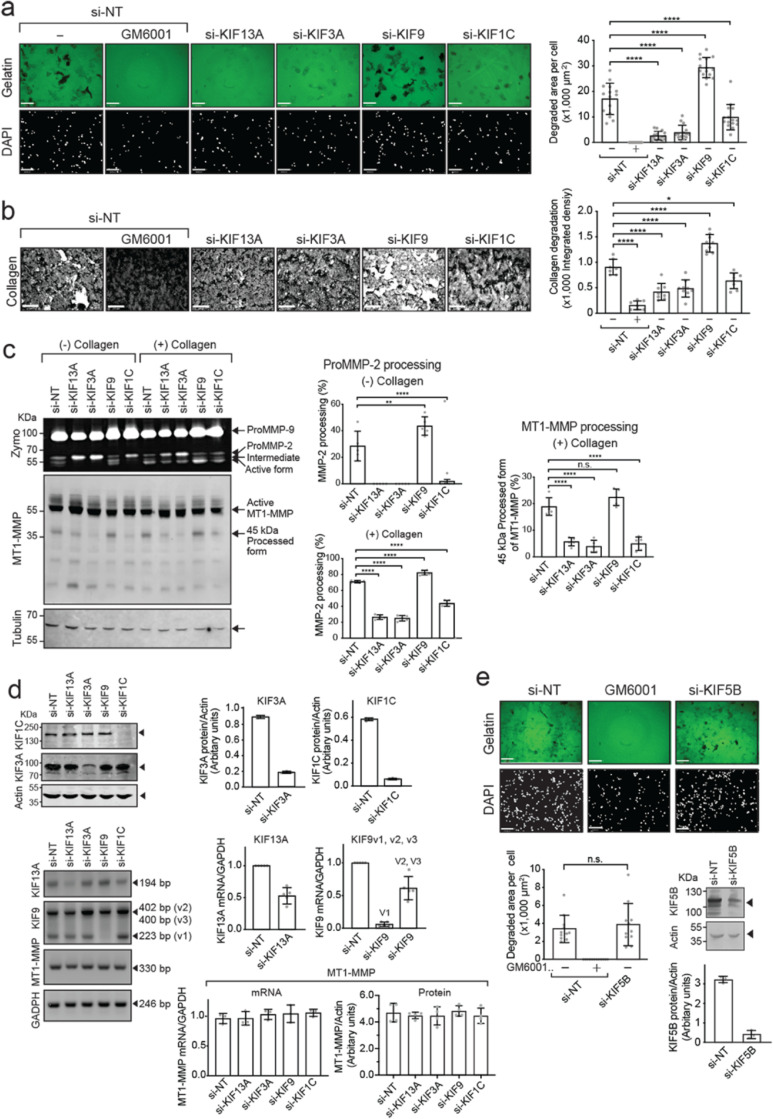
Knockdown of KIF13A, KIF3A, KIF9 and KIF1C alters MT1-MMP-mediated cell functions on the cell surface. **a.** HT-1080 cells were transfected with the indicated siRNAs and subjected to gelatin film degradation assay in the presence or absence of GM6001 (10 μM). Scale bars are 130 μm. Quantification of the degradation area (μm^2^) per cell in HT-1080 cells transfected with the indicated siRNAs was show as a graph (right panel). Data are presented as the mean of fifteen independent microscopic fields of view and are representative of five independent experiments. One-way ordinary Anova with Tuckey's multiple comparisons test. Data are shown as mean ± SD. ****p < 0.0001. **b.** HT-1080 cells were transfected with the indicated siRNAs and subjected to collagen film degradation assay in the presence or absence of GM6001 (10 μM). Scale bars are 130 μm. Quantification of the collagen layer's integrated density in HT-1080 cells transfected with the indicated siRNAs was shown as a graph (right panel). Data are presented as the mean of eight independent microscopic fields of view and are representative of five independent experiments. One-way ordinary Anova with Tuckey's multiple comparisons test. Data are shown as mean ± SD. ****p < 0.0001; *p < 0.05. **c.** HT-1080 cells transfected with the indicated siRNAs were cultivated in the presence or absence of collagen I (100 μg/ml) for 24 h. Culture media were analyzed by zymography (Zymo) for proMMP-2 activation. Cell lysates were subjected to Western blotting using antibodies for the MT1-MMP Hpx domain and tubulin. Arrows point bands of interest. ProMMP-2, 68 kDa proMMP-2; Intermediate, 65 kDa intermediate form; Active form, 62 kDa active MMP-2. Quantification of the percentage of MMP-2 processed forms over the total was shown as a graph (middle panel). The top panel shows processed MMP-2 without collagen stimulation, and the bottom panel shows the MMP-2 activation upon collagen stimulation. Quantification of the 45 kDa MT1-MMP processed forms (%) upon collagen stimulation was shown as a graph (right panel). Data are calculated from five independent experiments. One-way ordinary Anova with Tuckey's multiple comparisons test. Data are presented as a mean of five blots representative of five independent experiments. One-way ordinary Anova with Tuckey's multiple comparisons test. Data are shown as mean ± SD. ****p < 0.0001; **p < 0.01. n.s., non-significant. **d.** Efficiency of KIF knockdown was assessed by Western blotting for KIF1C and KIF3A and by RT-PCR for KIF13A, KIF9v1-v3. The effect of KIF knockdown on MT1-MMP mRNA level was also examined. Quantification data were shown as a graph. Western blot band intensities were normalized with Actin band intensities, and mRNA normalized with GAPDH. **e**. HT1080 cells transfected with siRNA for KIF5B were subjected to gelatin film degradation assay. The top panel shows representative images from each treatment as indicated. The bottom left panel shows quantitative data as degradation area per cell. Data are shown as mean ± SD (n=12). n.s., non-significant. The bottom right panel shows quantitative data of KIF5B measured by Western blot. Data are presented as a mean of three blots representative of three independent experiments.

Knockdown of KIF13A, KIF3A, and KIF1C notably decreased MT1-MMP-mediated gelatin film degradation, whereas the knockdown of KIF9 enhanced it in a statistically significant manner compared to non-targeting siRNA (si-NT) transfected cells (*p* < 0.0001) (Fig. 1a). Western Blotting for KIF3A and KIF1C and RT-PCR for KIF13A and KIF9 (Fig. 1d, top and middle panels) confirmed the efficiency of silencing. We used RT-PCR to confirm the knockdown of KIF13A and KIF9 because of the lack of suitable antibodies for Western Blotting. Interestingly, siRNA for KIF9 by smart pool siRNA (si-KIF9) was selective for KIF9-v1 (knocked down by more than 90%), as KIF9-v2 and -3 mRNA levels were knocked down only by 39%. We also confirmed that silencing any of these *kinesin* genes did not alter MT1-MMP mRNA or protein levels (Fig. 1d, bottom panel). Since it has been reported that KIF5B mediates MT1-MMP intracellular trafficking in primary macrophages and breast cancer cell line, MDA-MB231 [22,25,26], we also re-examined KIF5B knockdown in HT-1080 cells. The data showed that KIF5B knockdown did not affect gelatin film degradation (Fig. 1e). Next, we examined the effect of KIF knockdown on the collagenolytic activity of MT1-MMP in HT-1080 cells as the type I collagen represents its physiological substrate. As shown in Fig. 1b, KIF13A, KIF3A, and KIF1C knockdowns decreased collagen degradation by HT1080 cells, while the knockdown of KIF9 enhanced it in a statistically significant manner (Fig. 1b, right panel). These data indicated no differences between gelatin or collagen substratum regarding the role of these KIFs.

Another cell surface activity of MT1-MMP is proMMP-2 activation. ProMMP-2 activation can be induced by adding collagen I (100 μg/ml) in the culture medium [28]. We confirmed that the collagen-induced proMMP-2 activation is due to MT1-MMP in HT-1080 cells (Supplementary Fig. S1e, f). As indicated in Fig. 1c, the conditioned medium from si-NT-transfected cells, without collagen, showed proMMP-2 (ProMMP-2, 68 kDa) and its intermediate form (Intermediate, 65 kDa) (Fig. 1c, left panel). Silencing *Kif13a, Kif3a,* and *Kif1c* genes prevented the generation of the intermediate form, whereas silencing the *Kif9* gene accelerated proMMP-2 processing to its intermediate and active forms (Active form, 62 kDa, Fig. 1c, left panel). Upon collagen stimulation, around 71% of proMMP-2 was converted to its active form in si-NT-transfected cells (Fig. 1c, center lower panel). The knockdown of KIF13A and KIF3A significantly reduced the activation down to 26% and 25%, respectively. Knockdown of KIF1C seemed to moderately decrease it to 44% (Fig. 1c, center lower panel). Conversely, silencing the *Kif9* gene enhanced the activation to around 80% in a statistically significant manner (Fig. 1c, center lower panel). Interestingly, we noted that the generation of the 45 kDa processed form of MT1-MMP, which has been shown to coincide with functional activation of MT1-MMP, was also affected by KIF knockdown (Fig. 1c, left panel) [29]. The knockdown of KIF13A, KIF3A, and KIF1C significantly reduced the generation of the 45 kDa form (Fig. 1c, left and right panels), while the knockdown of KIF9 tends to increase it. However, the effect was statistically insignificant (Fig. 1c, left and right panels).

### Knockdown of KIF13A, KIF3A, KIF9, and KIF1C alters MT1-MMP localization at the cell-matrix interface

**Fig. 2:**
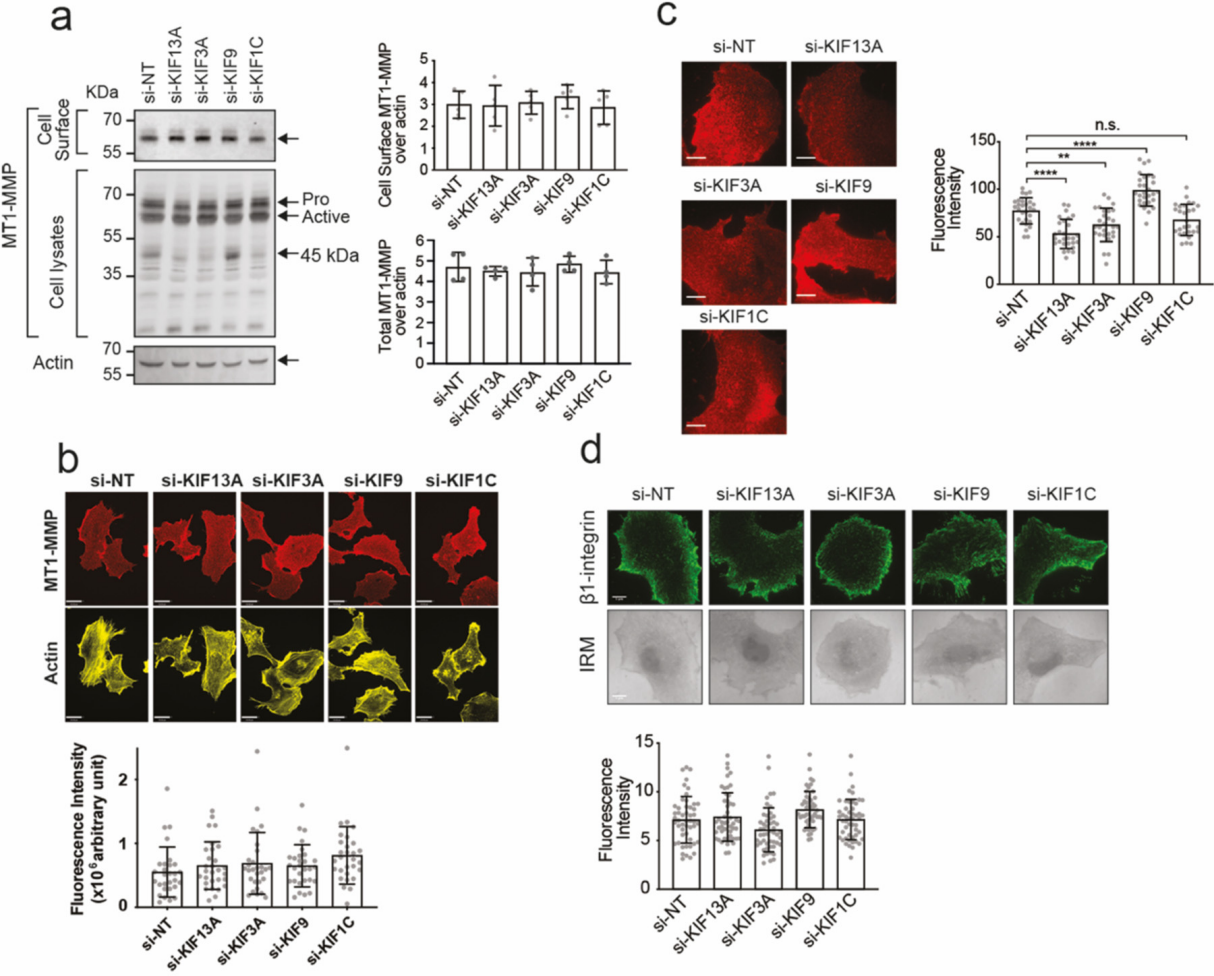
Knockdown of KIF13A, KIF3A, KIF9, and KIF1C modifies MT1-MMP localization at the substrate-attachment side. **a.** HT-1080 cells were transfected with the indicated siRNAs subjected to cell surface biotinylation. Proteins eluted from the streptavidin beads were analyzed by Western blot using an anti-Loop MT1-MMP antibody. Whole-cell lysates were analyzed by Western blot using 1D8 anti-MT1-MMP and anti-actin antibodies. Arrows point bands of interest. Quantification of the cell surface MT1-MMP in the left panel is shown as a graph (right panel). MT1-MMP band intensities were normalized by the actin band. Data are presented as mean ± SD. (n = 5) **b.** HT1080 cells transfected with siRNAs targeting KIFs were subjected to indirect immunofluorescent staining with anti-MT1-MMP antibody without permeabilization and imaged with confocal microscopy. Representative extended focus images of three independent experiments were shown for MT1-MMP (red) and actin (yellow) (top panel). Quantitative data were shown as a graph (bottom panel). Fluorescent intensity of extended focus image per cell was measured, and the data are presented as mean ± SD. **c.** HT-1080 cells transfected with siRNAs targeting the KIFs were stained with anti-MT1-MMP antibody without permeabilizing plasma membrane (red) and imaged by TIRF microscopy. Representative images of three independent experiments are shown (left panel). Scale bars are 10 μm. Quantitative data of the fluorescent intensities at the substrate-attachment side were shown as a graph (right panel). Data are presented as mean ± SD. (n = 30). ****p < 0.0001; **p < 0.01; n.s., non-significant. **d.** HT-1080 cells were transfected with siRNAs targeting the selected KIFs. After 72 h, cells were stained with anti-β1 integrin (green) antibody without permeabilization and imaged by TIRF microscopy. Representative images of three independent experiments are shown (top panel). Scale bars are 10 μm. Quantification of β1 integrin expression at the substrate attachment side in HT-1080 cells was shown as a graph (bottom panel). Data are presented as mean ± SD. (n=30).

The reduced or increased cell surface MT1-MMP activities upon knockdown of KIFs may be caused by the different levels of MT1-MMP on the cell surface. To investigate this possibility, we carried out cell surface biotinylation experiments. The data indicated that KIF knockdown did not affect the overall cell surface level of MT1-MMP (Fig. 2a). This result was confirmed by immunofluorescence staining of the cell surface MT1-MMP (Fig. 2b), showing no difference in cell surface MT1-MMP level upon knockdown of KIFs. In this experiment, we carried out immunocytochemistry without permeabilizing the plasma membrane, allowing us to stain only cell surface MT1-MMP. Therefore, we hypothesized that these KIF knockdown changed the localization of MT1-MMP at the cell-matrix interface without influencing the overall cell surface level. We then employed total internal reflection fluorescent (TIRF) microscopy to examine the levels of cell surface MT1-MMP at the ventral side of the membrane. As shown in Fig. 2c, silencing *Kif13a* and *Kif3a* genes significantly reduced MT1-MMP localization at the cell-matrix interface, whereas the knockdown of KIF9 increased it (Fig. 2c). Knockdown of KIF1C did not change MT1-MMP localization significantly (Fig. 2c). The knockdown of these KIFs did not influence cellular attachment to the gelatin, as it did not impact the level of cell surface β1 integrin (Fig. 2d) and close cell-matrix contacts detected by interference reflection microscopy (IRM) (Fig. 2d, top panel). These data indicate that knocking down KIF3a, KIF13a, and KIF9 altered MT1-MMP-dependent activity by changing the level of MT1-MMP localization at the cell-matrix interface.

### The knockdown of KIF13A, KIF3A, and KIF9 alters MT1-MMP-mediated cell invasion through a microporous membrane and MT1-MMP-dependent cell migration in 3D collagen

**Fig. 3:**
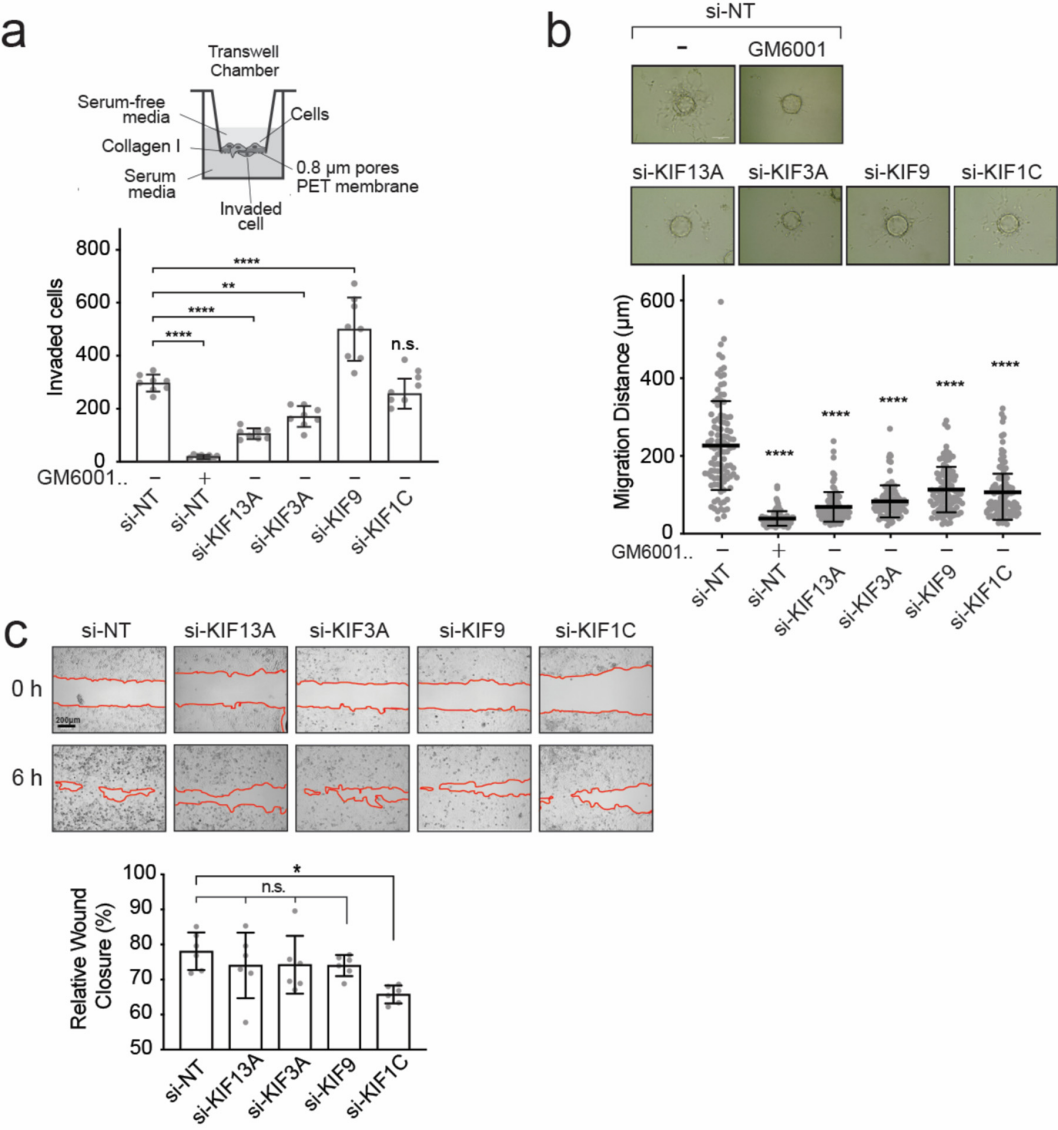
The knockdown of KIF13A, KIF3A, KIF9, and KIF1C affects MT1-MMP-dependent collagen invasion. **a.** HT1080 cells transfected with siRNA indicated were subjected to the same invasion assay, and data are shown as a graph (bottom right panel). Data are presented as the mean of the number of cells migrated through six trans-wells for each treatment and represent three independent experiments. **** *p* < 0.0001, ** *p* < 0.001, n.s., non-significant. **b.** HT1080 cells were transfected with siRNAs targeting the selected KIFs and subjected to bead invasion assay in the presence or absence of GM6001 (10 μM). A representative image of cells migrating from the bead's surface into collagen type-I matrix (2mg/ml) is shown (top panel). Scale bar,130 μm. Data are presented as the mean of the distance migrated by one hundred cells for each treatment. Error bars SD, **** *p* < 0.0001, n.s., non-significant. **c.** HT1080 cells were transfected with siRNAs targeting the selected KIFs and subjected to wound healing assay on plastic. Images of 0h and 6h time points are shown. Images are representative of three independent experiments. Red lines highlight the migration front. Scale bar, 200 μm. Quantification of wound healing assay data was shown as a graph (bottom panel). Data are presented as the mean percentage of wound closure relative to the initial gap area. * *p* < 0.019.

We next examined the effect of KIF knockdown on MT1-MMP-dependent cell invasion. We employed two different invasion assays: collagen invasion assay using trans-well chambers coated with collagen gel (Fig. 3a) and microcarrier bead invasion assay that assesses migration distance of cells from the beads’ surface within 3D collagen gel (Fig. 3b). The cellular invasion in these assays depends entirely on endogenous MT1-MMP in HT-1080 cells (Supplementary Fig S1f, g). As shown in Fig. 3a (bottom right panel), the knockdown of KIF13A and KIF3A significantly decreased the invasion, whereas KIF9 knockdown significantly enhanced it in the trans-well invasion assay. KIF1C knockdown did not affect the cell invasion (Fig. 3a, bottom right panel). In the microcarrier bead invasion assay (Fig. 3b), the knockdowns of KIF13A, KIF3A, and KIF1C significantly reduced cell migration. Interestingly, si-KIF9-transfected cells also migrated significantly less (Fig. 3b), contrary to the trans-well invasion assay (Fig. 3a). However, the effects of KIF knockdown may potentially be attributed to the influence on vesicle transport of other molecules critical for the cell migration machinery. To test this possibility, we examined the effect of knockdown of KIF13A, KIF3A, and KIF9 on cell migration on a plastic surface by employing a scratch wound healing assay (Fig. 3c). The data indicate that the knockdown of KIF13A, KIF3A, and KIF9 did not influence the cell migration (Fig. 3c). On the other hand, KIF1C knockdown slightly decreased it (P = 0.019), indicating that KIF1C may be involved in the general cell migration (Fig. 3c).

### KIF13A and KIF3A colocalize with MT1-MMP-containing vesicles

**Fig. 4:**
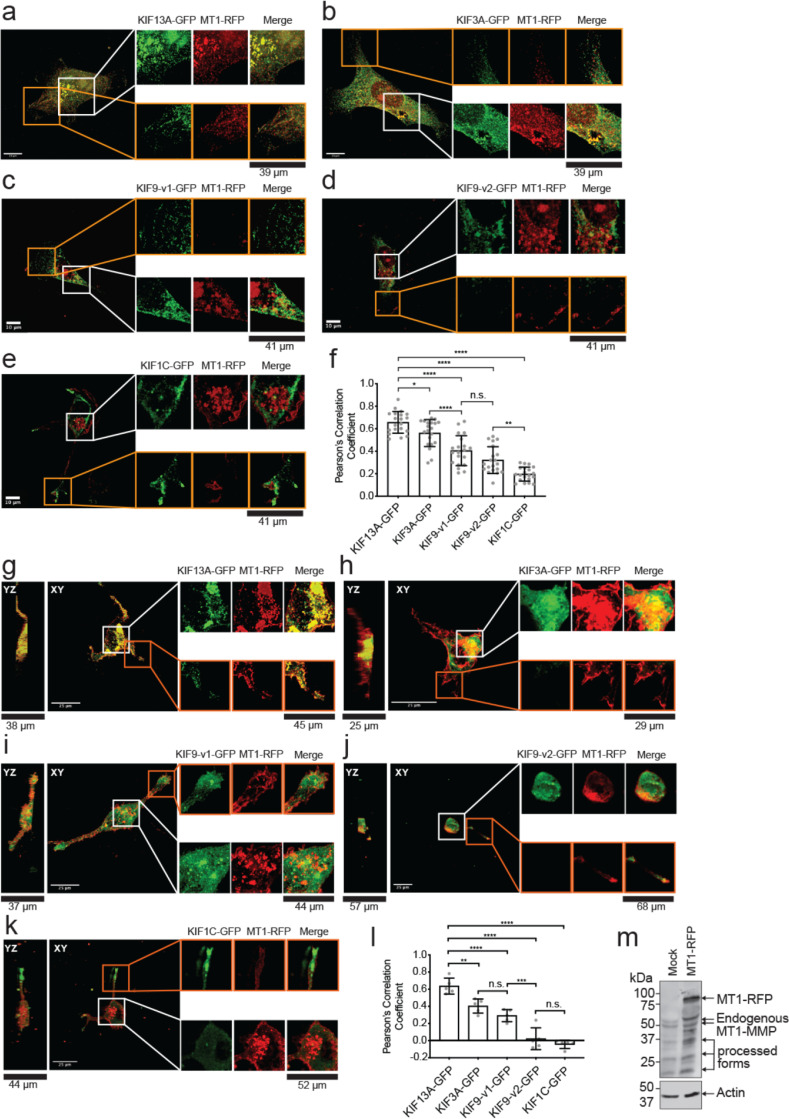
MT1-RFP and KIF-GFP colocalization in HT-1080 cells cultured on the gelatin-coated cover glass and within the 3D collagen matrix. **a, b, c, d, e.** HT-1080 cells transfected with GFP-tagged KIFs (indicated, green) and MT1-RFP (red) were cultured on the gelatin-coated cover glass. Extended focus representative images are shown. White boxes enclose enlarged areas for the perinuclear region and orange boxes for the periphery region. Scale bars indicate 10 μm. **f.** Quantification of colocalization between GFP-tagged KIFs and MT1-RFP. Data are presented as the mean of 20 PCC calculated for 20 different cells per treatment and represent three independent experiments. One-way ordinary Anova with Tuckey's multiple comparisons test. Data are shown as mean ± SD. ****p < 0.0001: *p < 0.05; **p < 0.01; n.s., non-significant. **g, h, i, j, k.** HT-1080 cells transfected with GFP-tagged KIFs (green) and MT1-RFP (red) were cultured within 3D collagen gel and imaged by confocal microscopy. Representative extended focus images and the corresponding orthogonal views are shown. White boxes enclose enlarged areas. Scale bars are 25 μm. **l.** Quantification of colocalization between GFP-tagged KIFs and MT1-RFP. Data are presented as the mean of five PCCs calculated for five different cells per treatment and represent three independent experiments. One-way ordinary Anova with Tuckey's multiple comparisons test. Data are presented as mean ± SD; **p < 0.01; ***p < 0.001; ****p < 0.0001; n.s., non-significant. **m**. Western blot of HT1080 cells transfected with empty vector (Mock) and MT1-RFP plasmid. Anti-MT1-MMP Hpx domain (222-1D8) was used to visualize MT1-MMP (top). Actin was used as a loading control (bottom). Note that endogenous MT1-MMP bands were also shown in addition to MT1-RFP.

Next, the cellular distribution of MT1-RFP and GFP-tagged KIF13A, KIF3A, KIF9-v1, KIF9-v2, and KIF1C were analyzed. As shown in Fig. 4a, KIF13A-GFP co-localized with MT1-RFP and the two proteins exhibited a similar distribution pattern within the perinuclear (white box) and the cell periphery areas (orange box). KIF3A-GFP also colocalized with MT1-RFP-positive vesicles within the perinuclear region (Fig. 4b, white box). However, the two proteins did not colocalize at the cell periphery (Fig. 4b, orange box). KIF9-v1-GFP colocalized with MT1-RFP within the perinuclear region (Fig. 4c, White box), but not the cell periphery (Fig. 4c, orange box). KIF9-v2-GFP- and KIF1C-GFP-transfected cells did not show a distinct vesicular-like distribution of MT1-RFP. Also, KIF9-v2-GFP and KIF1C-GFP did not colocalize with MT1-RFP in the perinuclear nor cell periphery region (Fig.4d and e). It was interesting to note that KIF1C-GFP strongly accumulated at the tips of the trailing edges (Fig. 4e, orange box). Pearson's correlation coefficients (PCCs) confirmed that KIF13A-GFP and KIF3A-GFP had the highest degree of colocalization (Fig. 4f). On the other hand, KIF9-v2-GFP and KIF1C-GFP exhibited the least colocalization with MT1-RFP. These cells were also embedded in a 3D collagen matrix and imaged by confocal microscopy (Fig. 4g-i). The data confirmed that KIF13A-GFP exhibited the highest colocalization with MT1-RFP (Fig. 4g, i), whereas KIF3A-GFP colocalization was found only within the perinuclear region (Fig. 4h, i). Under these conditions, we confirmed no colocalization of KIF9-v2-GFP and KIF1C-GFP with MT1-RFP (Fig. 4j–i). We also confirmed that MT1-RFP expressed in HT1080 cells is intact, indicating that the RFP signal detected in the cells reflect the functional enzyme (Fig. 4m).

### KIF13A, KIF3A, and KIF9-v1 transport MT1-MMP-containing vesicles

**Fig. 5:**
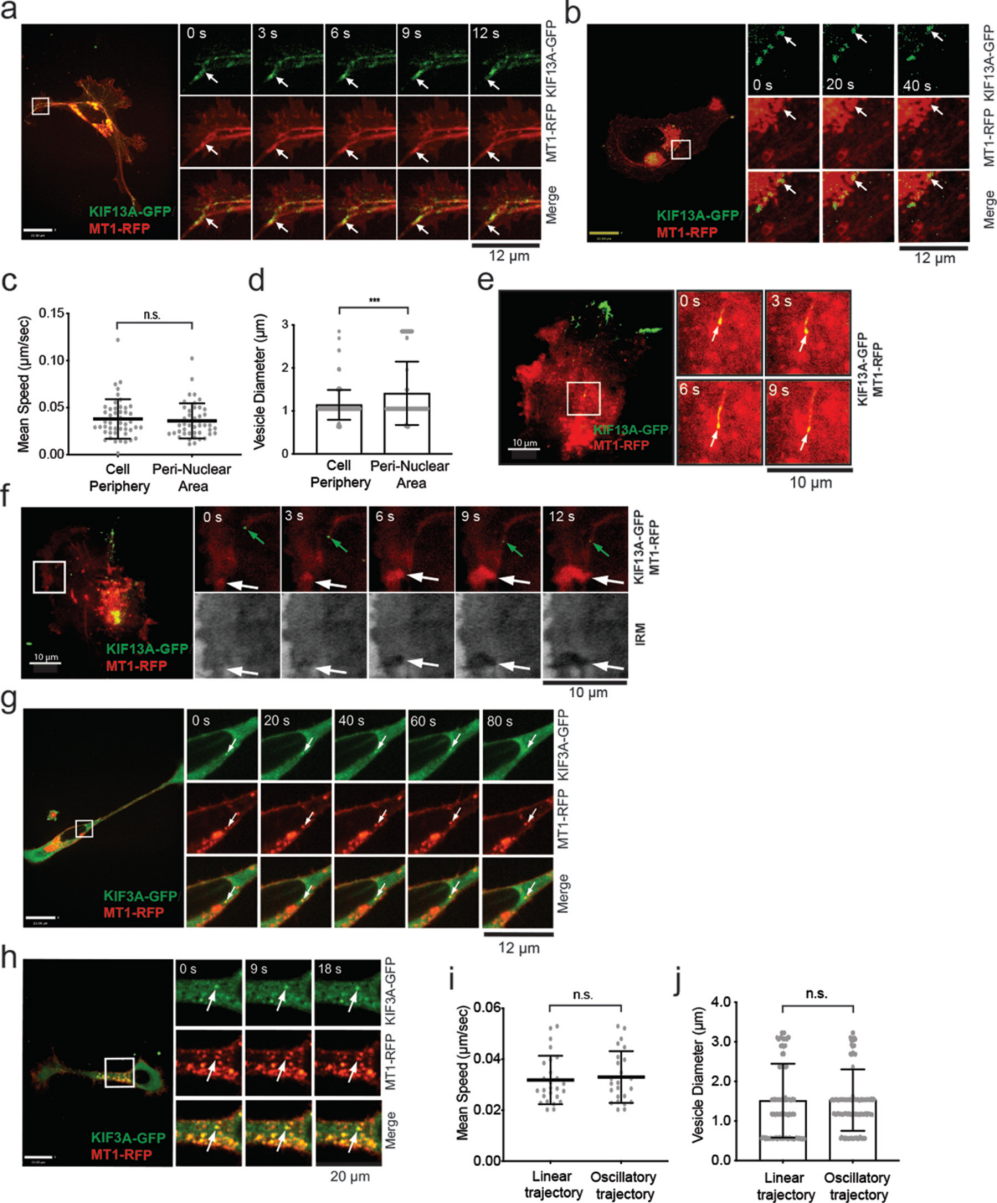
MT1-RFP was co-trafficked with KIF13A-GFP and KIF3A-GFP **a, b.** HT-1080 cells were transfected with KIF13A-GFP (green) and MT1-RFP (red), and they were subjected to live-cell imaging on the gelatin film by confocal microscopy. Representative image time sequences are shown. White arrows point vesicles of interest. Scale bars are 22 μm. **c**. Quantification of the mean velocity double-positive vesicles for MT1-RFP and KIF13A-GFP at the leading edge and nuclear area. Representatives of five independent experiments are shown, and data are calculated for 47 and 44 trajectories for the cell edge and the peri-nuclear area, respectively. Unpaired T-test with Welch's correction. Data are shown as mean ± SD. n.s., non-significant. **d.** Quantification of double-positive vesicle diameter at the cell edge and peri-nuclear area. Data represent five independent experiments and are calculated for 129 vesicles for the leading edge and the peri-nuclear area. Data are shown as mean ± SD. *** *p* < 0.001. **e, f.** HT-1080 cells transfected with KIF13A-GFP (green) and MT1-RFP (red) were subjected to live-cell imaging by TIRF microscopy. Representative image time sequences are shown. White arrows point vesicles of interest. IRM, internal reflection microscopy. The scale bar is 10 μm. **g, h**. HT-1080 cells transfected with KIF3A-GFP (green) and MT1-RFP (red) were subjected to live-cell imaging on the gelatin film by confocal microscopy. Representative image time sequences are shown. White arrows point vesicles of interest. Scale bars are 22 μm. **i.** Quantification of the mean velocity of double-positive vesicles for MT1-RFP and KIF3A-GFP with linear or oscillatory trajectories. Data represent five independent experiments and are calculated for 26 and 24 linear and oscillatory trajectories. Unpaired T-test with Welch's correction. Data are shown as mean ± SD. n.s., non-significant. **j.** Quantification of double-positive vesicle diameter. Data represent five independent experiments and are calculated for 129 vesicles for each group. An unpaired T-test with Welch's correction was used. Data are shown as mean ± SD. n.s., non-significant.

These cells were next subjected to time-lapse imaging using confocal microscopy. The expression of KIF13A-GFP often made a distribution of MT1-RFP signals into a tubular-like shape, extending from the perinuclear regions towards the tips of the ruffling membrane (Fig. 5a). Numerous KIF13A-GFP signals were observed moving along these MT1-RFP tubular-like structures, accumulating at the cell periphery. Therefore, the tubular-like structures are microtubules, and MT1-RFP is distributed over the microtubules (*movie S1*). Vesicles double-positive for KIF13A-GFP and MT1-RFP could also be detected within the perinuclear area. Despite MT1-RFP strongly accumulated within this region, it was possible to observe KIF13A-GFP transporting MT1-RFP-containing vesicles (Fig. 5b, *movie S2).* No significant differences were observed in the velocity or size of vesicles between the cell periphery or within the perinuclear area (Fig. 5c, d).

We also carried out live-cell imaging on TIRF microscopy (Fig. 5e, *movie S3*), and the vesicles positive for KIF13A-GFP and MT1-RFP were detected at the cell-matrix interface. Combining time-lapse IRM with TIRF microscopy, we also observed that MT1-RFP progressively accumulated at the growing adherent membrane on a gelatin-coated glass surface (Fig. 5f). As discussed above, MT1-RFP-positive vesicles were again distributed in tubular-like structures, which pointed toward the growing highly adherent membrane (Fig. 5f, *movie S4)*

Expression of KIF3A-GFP in HT-1080 cells did not affect the shape of MT1-RFP-positive vesicles (Fig. 5g, h). The vesicles positive for KIF3A-GFP and MT1-RFP were either moving linearly (Fig. 5g, *movie S5*) or oscillatory within the perinuclear region (Fig. 5h, *movie S6*). Their mean velocities and diameters were equal, regardless of their trajectory (Fig. 5i, j).

Vesicles double-positive for KIF9-v1-GFP and MT1-RFP were detected within the perinuclear area of HT-1080 cells (Supplementary Fig S3a). These vesicles did not move, but their fluorescence intensity faded over time, suggesting that the vesicles moved out of the focus plane, along the z-axis, most likely towards the dorsal side of the cell. No vesicles positive for both KIF9-v2-GFP and MT1-RFP could be detected, as shown in Supplementary Supplementary Fig S3b. Likewise, we could not observe any MT1-RFP-positive vesicles trafficked by KIF1C-GFP (Supplementary Fig S3c). Once more, KIF1C-GFP was accumulated at the trailing edge (Supplementary Fig. S3c).

### Collaboration of KIF13A and KIF3A to transport MT1-MMP vesicles to the leading edge

**Fig. 6:**
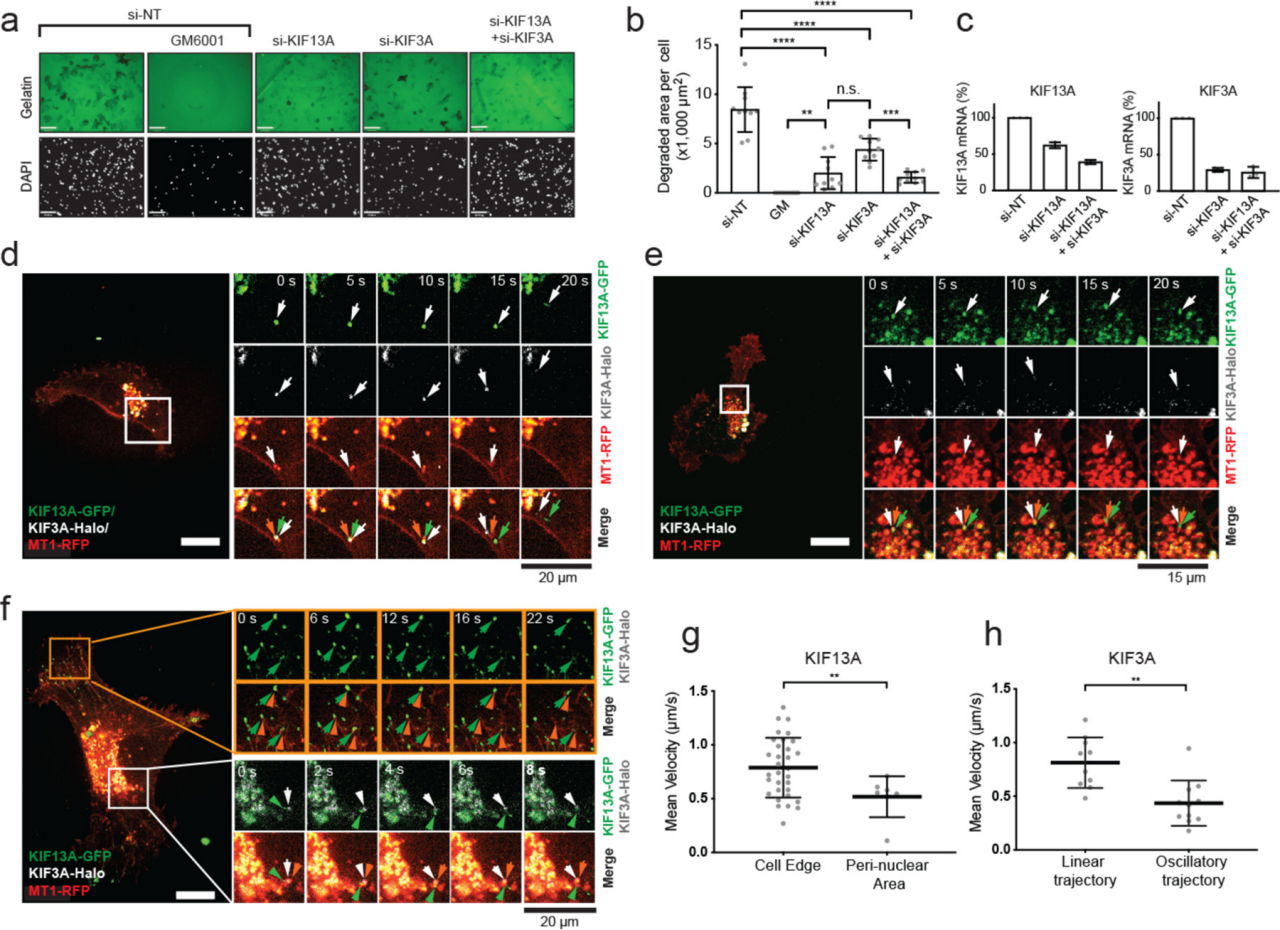
KIF13A and KIF3A transport MT1-MMP-containing vesicles. **a.** HT-1080 cells were transfected with siRNAs targeting the selected KIFs and subjected to gelatin film degradation assay in the presence or absence of GM6001 (10 μM). Scale bars are130 μm. **b.** Quantification of the degradation area (μm^2^) per cell in HT1080 cells transfected with the specified siRNAs. Data are presented as a mean of 10 independent microscopic fields of view and are representative of three independent experiments. One-way ordinary Anova with Tuckey's multiple comparisons test. Data are shown as mean ± SD. ****p < 0.0001; ***p < 0.001; **p < 0.01; n.s., non-significant. **c.** Efficiency of KIF knockdown was assessed by one-step RT-PCR. Quantification of KIF13A and KIF3A mRNA fold changes relative to GADPH mRNA. The data are representative of three independent experiments. **d, e, f.** HT-1080 cells were transfected with KIF13A-GFP (green), KIF3A-GFP (white), and MT1-RFP (red), and they were subjected to live-cell imaging by confocal microscopy. Representative image time sequences are shown. Arrows point vesicles of interest. Scale bars are 22 μm. **g.** Quantification of mean velocities of MT1-RFP-containing vesicles transported by KIF13A-GFP at the cell edge and nuclear area. Data are representative of three independent experiments and are calculated for 32 and seven trajectories for the cell edge and the nuclear area, respectively. Unpaired T-test with Welch's correction. Data are shown as mean ± SD. **p < 0.01; n.s., non-significant. **h.** Quantification of mean velocities of MT1-RFP-containing vesicles transported by KIF13A-GFP and KIF3A-GFP with linear or oscillatory trajectories. Data are representative of three independent experiments and are calculated for 10 linear and 11 oscillatory trajectories. Unpaired T-test with Welch's correction. Data are shown as mean ± SD. **p < 0.01, n.s., non-significant.

We next investigated if KIF13A and KIF3A transport MT1-MMP vesicles independently or on the same pathway. HT-1080 cells transfected for si-KIF13A, si-KIF3A, and a combination of the two were subjected to gelatin film degradation (Fig. 6a, b, c). If these KIFs work independently, double knockdown of KIF3A and KIF13A should show additive inhibitory effects. However, if they work in the same pathway, such an effect should not be observed. Upon KIF13A knockdown, gelatin film degradation was decreased by around 78%, while KIF3A knockdown by 49%. When the two knockdowns were combined, gelatin film degradation was reduced by 82%, the same level as KIF13A knockdown alone (Fig. 6a, b). These data suggest that these two motor proteins function on the same pathway to deliver MT1-MMP to the cell surface.

Next, HT-1080 cells expressing KIF13A-GFP, KIF3A-HaloTag, and MT1-RFP were subjected to time-lapse imaging. Vesicles positive for all three signals were detected within the perinuclear region (Fig. 6d, e). As pointed by the arrows colored according to the different channels, these vesicles moved from the perinuclear area towards the cell periphery (Fig. 6d). KIF13A-GFP and KIF3A-HaloTag co-localized with MT1-RFP-, containing vesicle until MT1-RFP fluorescence signal had faded, indicating that the MT1-RFP vesicle had fused with the plasma membrane (Fig. 6d). Then, KIF13A-GFP and KIF3A-HaloTag independently trafficked back towards the center of the cell (Fig. 6d). In other cases, these vesicles positive for KIF13A-GFP, KIF3A-HaloTag, and MT1-RFP were oscillating within the perinuclear region (Fig. 6e). On the other hand, vesicles at the cell periphery were positive for only KIF13A-GFP and MT1-RFP (Fig. 6f, *movie S7*). These vesicles were either moving within the cell edge or the perinuclear region. Interestingly, with KIF3A-HaloTag expression, KIF13A-GFP- and MT1-RFP-positive vesicles moved significantly faster at the cell edge within the perinuclear area (Fig. 6g), compared with expression of KIF13A-GFP and MT1-RFP (Fig. 5c). With KIF13A-GFP expression, KIF3A-HaloTag- and MT1-RFP-positive vesicles moved significantly faster when proceeding with a linear trajectory than when they were oscillating (Fig. 6h), compared with expression of KIF3A-GFP and MT1-RFP (Fig. 5i). Taken together, we concluded that KIF13A and KIF3A co-traffic MT1-MMP-containing vesicles around the perinuclear areas, and KIF13A takes over the vesicles at the periphery to traffic them towards the plasma membrane of the cell.

Given the role of KIF3A and KIF13A in MT1-MMP vesicle transport to degrade matrix, increased matrix-degrading activity upon KIF9 knockdown may be due to increased KIF3A and KIF13A-mediated vesicle transport of MT1-MMP. To test this hypothesis, *Kif9* was co-silenced with *Kif3a* or *Kif13a.* As shown in Supplementary Fig S4, increased gelatin film degradation by KIF9 knockdown was significantly decreased upon co-silencing *Kif3a or Kif13a* genes. These data suggest that knockdown of KIF9 made MT1-MMP vesicles available for KIF3A- and KIF13A-dependent vesicle transport, resulting in increased matrix degradation.

We next carried out live-cell imaging of HT1080 cells expressing MT1-RFP and knocked down for KIF3A, KIF13A, and KIF9 (movie S8-S11). In non-targeting control cells, MT1-RFP vesicles were actively transported from the perinuclear area to the cell periphery (movie S8). When KIF3A or KIF13A was knocked down, the lateral vesicle transports from the perinuclear region to the cell periphery were completely absent (movies S9, S10). Upon KIF9 knockdown, MT1-RFP vesicles were larger and actively transported (movie S11). The data suggest that KIF13A-dependent vesicle transport to the cell periphery needs KIF3A and KIF13A-dependent vesicle transport in the perinuclear region.

**Fig. 7:**
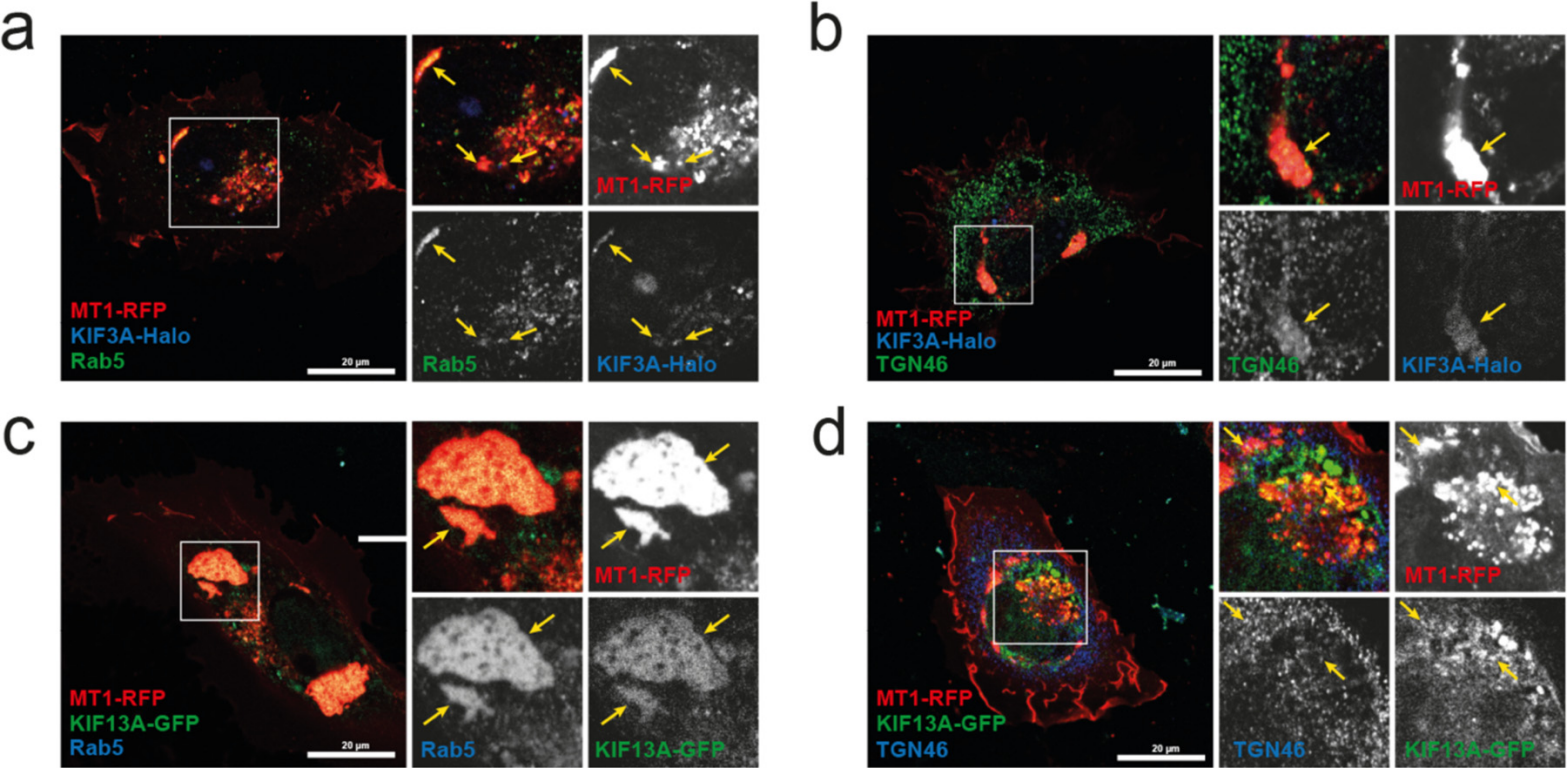
Localisation of MT1-RFP, KIF3A-Halo, and KIF13A-GFP at the endosomes and the trans-Golgi Cells co-expressing MT1-RFP and KIF3A-Halo (**a, b**), MT1-RFP and KIF13A-GFP (**c, d**) were stained for endosome marker, Rab5 (**a, c**), or trans-Golgi marker, TGN46 (**b, d**). Boxed area in the left image was enlarged and each channel and merged images were shown on the right. KIF3A-Halo was visualized by reacting with Halo-ligand Coumarin (**a, b**). Yellow arrows indicate colocalised signals of three channels (**a, b, c, d**). Single section images were acquired with Zeiss LSM 980 with Airyscan2 in super resolution mode.

To define the detailed location of MT1-MMP and KIFs in the perinuclear area, we next investigated the relative location of MT1-RFP, KIF3A-Halo, and KIF13A-GFP with endosomes and trans-Golgi (Fig. 7). It has been shown that KIF3A is involved in the post- Golgi transport of N-cadherin/β-catenin [30]. KIF13A has been shown to mediate vesicle transport from the endosomal recycling compartment to the plasma membrane [24,31,32]. As an endosome marker, we used Rab5. It has been reported that Rab5 regulates vesicle trafficking from the plasma membrane to the early endosomes and to mediate homotypic fusion of these compartments [33,34]. As a trans-Golgi marker, we chose TGN46 as it is a well-established *trans-Golgi* marker [35]. The cells expressing MT1-RFP and KIF3A-Halo, Rab5, and MT1-RFP colocalized well, and KIF3A-Halo partially localized (Fig 7a, yellow arrows). TGN46 signal was found to colocalize with KIF3A-Halo at the large organelles (Fig 7b, yellow arrows). In the cells expressing MT1-RFP and KIF13A-GFP, they were extensively colocalized at Rab5-positive large organelles, early endosomes (Fig. 7c, yellow arrows). KIF13A-GFP also colocalized with TGN46 partially (Fig. 7d, yellow arrows). These data indicate that MT1-MMP, KIF3A and KIF13A associate with both trans-Golgi and endosomes, and both KIF3A and KIF13A are likely to be responsible to transport MT1-MMP-containing vesicles from *trans*-Golgi to endosomes.

### Localization of MT1-MMP at focal adhesion (FA)

**Fig. 8:**
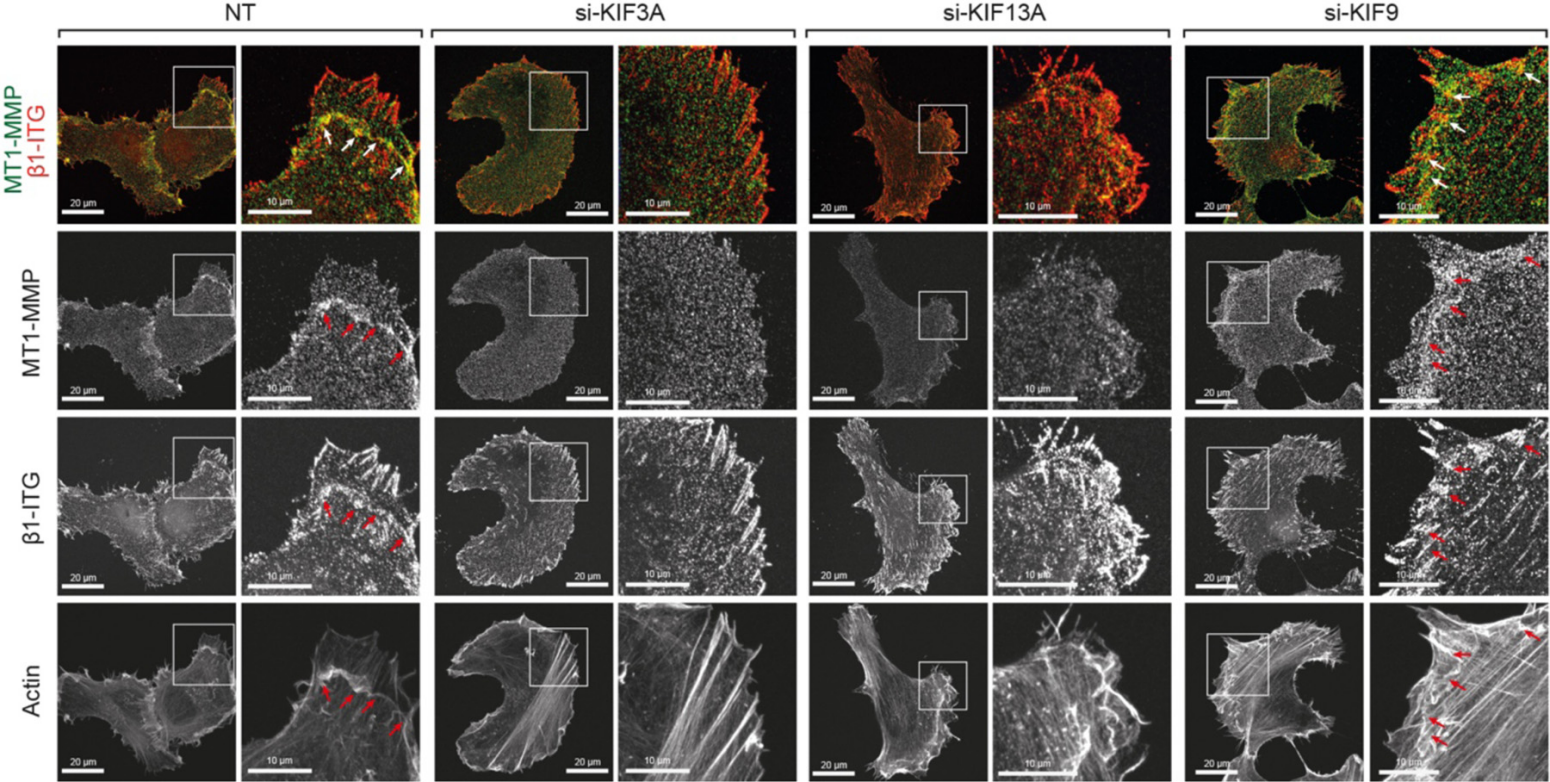
Effect of KIF knockdown on Localization of MT1-MMP at focal adhesion HT1080 cells transfected with siRNA for non-targeting (NT), KIF3A, KIF13A, and KIF9 were stained for cell surface MT1-MMP and β1-ntegrin subunit without permeabilizing cells. Arrows indicate where MT1-MMP signals colocalized with β1 integrins. Ventral surface images were acquired with Zeiss LSM 980 with Airyscan2 in super resolution mode. The ventral surface images were obtained by combining 2-4 sections (0.13 µm sections) images using orthogonal projection.

We and others have previously reported that MT1-MMP is localized at FA, where the distance of the plasma membrane and the matrix is the closest to degrade substratum in HT1080 cells [20,36]. Since KIF knockdown affects the matrix degradation, we examined the localization of endogenous MT1-MMP and β1 integrin subunit at the ventral cell surface upon KIF knockdown (Fig. 8). β1-integrin-positive FA signals were observed as large spots, and MT1-MMP and FA signals were unevenly distributed across the ventral surface in non-targeting siRNA-transfected cells (Fig. 8, NT). More FA spots were found towards the leading edge of cells, where MT1-MMP and high actin signals are colocalized (arrows). Upon KIF3A knockdown (si-KIF3A), the MT1-MMP signal distributed evenly, and it was no longer associated with FA and actin. Upon KIF13A knockdown (si-KIF13A), the overall MT1-MMP signal seems to be decreased at the ventral surface, and its associations with FA and actin were minimal. In cells with KIF9 knockdown (si-KIF9), distribution of MT1-MMP and FA spots were polarised, especially towards the leading edge of cells, like NT cells, where they colocalized (arrows). These data suggest that KIF3A and KIF13A-dependent MT1-MMP vesicle trafficking plays a crucial role in localizing MT1-MMP at FA.

### Genomic alterations of *Kif13a* and *Kif9* genes across The Cancer Genome Atlas (TCGA) studies

We next investigated if the expression level of KIF3A, KIF13A, and KIF9 correlates with cancer progression. Gene expression of KIF13A, KIF3A, and KIF9 across The Cancer Genome Atlas (TCGA) PanCancer Atlas studies were analyzed. Amplifications and deep deletions of *Kif13a, Kif3a,* and *Kif9* genes across the TCGA database were searched on cBioPortal (http://cbioportal.org) [37,38]. The term amplification indicates a high copy-number level per gene. Deep deletion implies a profound loss in the copy-number level per gene and often refers to a homozygous deletion. As shown in Fig. 10a, the *Kif13a* gene was amplified in 1.3% of samples across the TCGA PanCancer Atlas studies and deleted in 0.2%. On the other hand, the *Kif9* gene was amplified in only 0.1% of samples and deleted in 0.5%. These gene alterations were distributed across several cancer types of TCGA (Supplementary Fig S6b, c). More than seven in 100 women diagnosed with ovarian serous cystadenocarcinoma presented a *Kif13a* gene amplification (Supplementary Fig S6b). *Kif13a* gene was also amplified in more than 6% and 4% of patients diagnosed with bladder urothelial cancer and diffuse large-B cell lymphoma (DLBC), respectively (Supplementary Fig S6b). *Kif9* gene was deleted in over 6 out of 100 patients diagnosed with DLBC and in almost 3% of patients with kidney renal clear cell carcinoma (ccRCC) (Supplementary Fig. S6c). Next, we used the Pathology Atlas (https://www.proteinatlas.org/humanproteome/pathology) [39] to investigate whether KIF13A and KIF9 are considered prognostic markers for specific cancer types. We performed a Kaplan-Meier survival analysis of liver cancer patients with low or high mRNA expression of the *Kif13a* gene through the Pathology Atlas. We found that patients with high expression of the *Kif13a* gene had a significantly lower survival rate (Supplementary Fig.S 6d). We also performed the same analysis of renal and colorectal cancer patients with low or high mRNA expression of the *Kif9* gene. We found that patients with low expression had a significantly lower survival rate (supplementary Fig.S 6e, f).

*Kif3a* gene was amplified in 0.5% of cases across the TCGA PanCancer Atlas studies and deleted in 0.3% of them (Supplementary Fig S7a). 5% of patients with ccRCC presented an amplification of the *Kif3a* gene. However, we could not assess whether this gene alteration was associated with a change to the overall patients’ survival (supplementary Fig.S 7b). According to our search on the Pathology Atlas, *Kif3a* gene alterations are not considered prognostic markers for different cancer types.

## Discussion

**Fig. 9:**
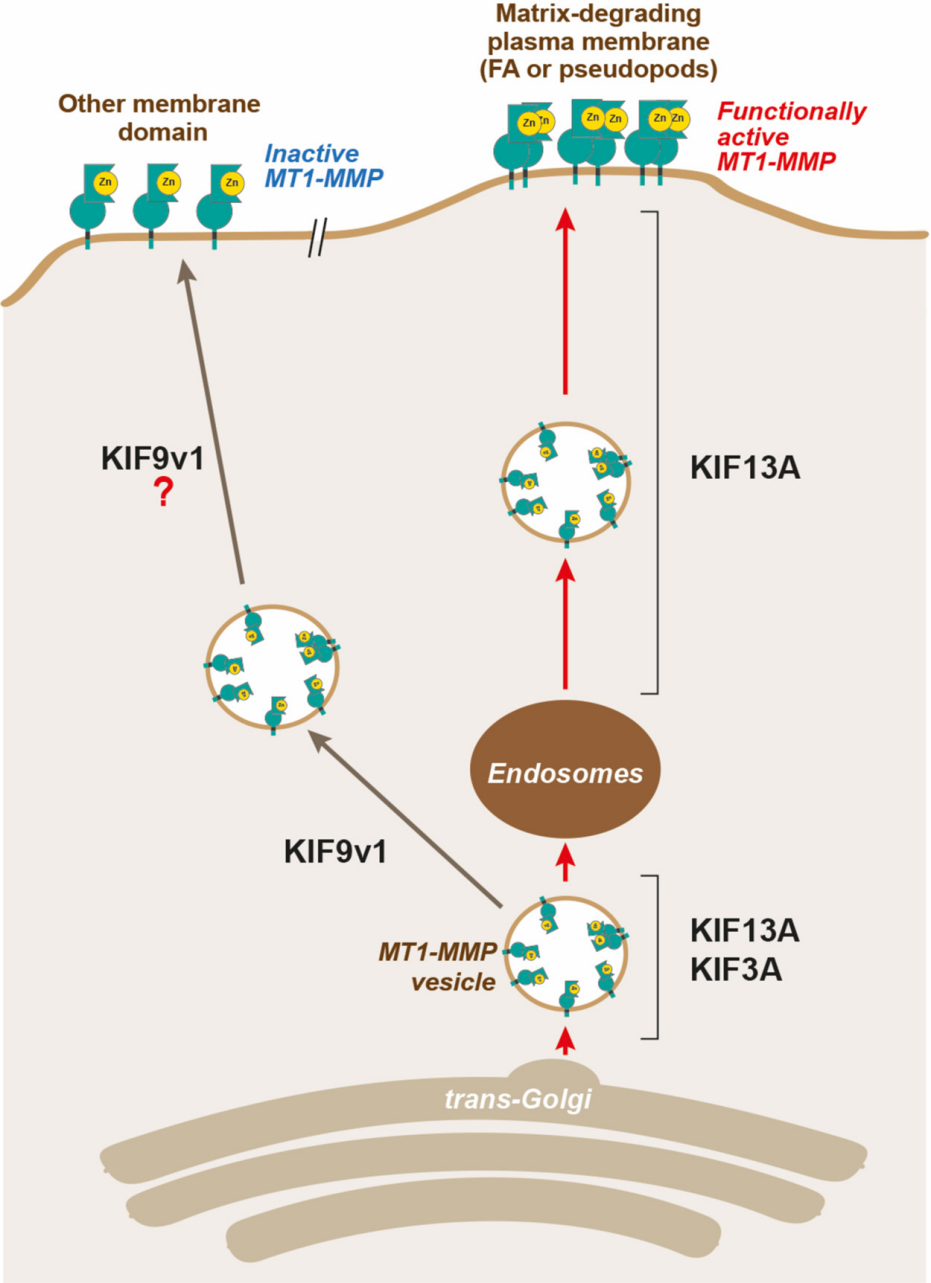
Possible involvement of KIF3A, KIF13A, and KIF9v1 in MT1-MMP-containing vesicle trafficking MT1-MMP containing vesicles are trafficked from the *trans*-Golgi to the endosomes by the cooperation of KIF3A and KIF13A. MT1-MMP is temporally stored in the endosomes, and KIF13A alone further traffics MT1-MMP vesicles from the endosomes to the plasma membrane. Without KIF3A, KIF13A cannot traffic the vesicles from the trans-Golgi to endosomes; thus, no MT1-MMP vesicles are transported from the endosomes to the plasma membranes. KIF3A/KIF13A-mediated MT1-MMP vesicle transport is destined to the leading edge of the plasma membrane, where MT1-MMP forms functionally active homodimers. KIF9v1 seems to traffic MT1-MMP vesicles to other membrane domains, where MT1-MMP is functionally inactive.

When invading cancer cells encounter a physical ECM barrier with narrow gaps of less than 7 μm in diameter, they utilize MT1-MMP to break through the barrier and create a path for migration [40]. In this process, cells localize MT1-MMP to the invading edge by directly trafficking MT1-MMP-containing vesicles along microtubules. Here, we have identified three kinesin motor proteins, KIF3A, KIF13A, and KIF9v1, directly involved in trafficking MT1-MMP-containing vesicles to the leading edge. Based on the findings in this study, we depicted a model of MT1-MMP vesicle transport by KIF3A, KIF13A, and KIF9v1 in Fig. 9. We discuss the evidence of each step below.

Although knockdown of KIF1C significantly reduced gelatin and collagen film degradation like KIF3A and KIF13A knockdown, KIF1C was unlikely to be directly involved in MT1-MMP vesicle transport. MT1-RFP and KIF1C-GFP did not colocalize when cells were cultured on a gelatin film or within a 3D collagen gel. There was a strong tendency of KIF1C to localize at the trailing edge of migrating cells rather than at the leading edge. A similar conclusion was made by Weisner *et al.* [22]. They observed that KIF1C knockdown decreased MT1-MMP-dependent gelatin film degradation at podosomes, but KIF1C did not colocalize with MT1-MMP vesicles. KIF1C may affect MT1-MMP vesicle transport indirectly, by potentially influencing cell migration machinery, as knockdown of KIF1C, but not other KIFs, reduced cell migration on plastic significantly (Fig. 3c), which is an MT1-MMP-independent process. Therefore, KIF3A and KIF13A are the major KIFs directly traffic MT1-MMP vesicles to the leading edge in HT1080 cells.

Interestingly, the same effect of knockdown of KIF3A, KIF13A, and KIF9 was also observed for proMMP-2 activation activity: knockdown of KIF3A or KIF13A inhibited collagen-induced proMMP-2 activation, and KIF9 knockdown enhanced activation. The data suggest that KIF3A and KIF13A-mediated vesicle transport is necessary for proMMP-2 activation by MT1-MMP. We and others have previously reported that the homodimer formation of MT1-MMP is essential for proMMP-2 activation on the cell surface [5,18,41,42], and that homodimerization of MT1-MMP occurs consistently at the leading edge of invading cells [43]. Thus, the membrane domain where KIF3A and KIF13A transport MT1-MMP allows the enzyme to form a homodimer, and MT1-MMP in other membrane domains is likely to be a non-functional monomer (Fig. 9). This hypothesis seems to make sense as proMMP-2 activation should occur at the leading edge where cells degrade ECM to invade into tissue.

*Trans*-Golgi is the last station of the secretory pathway of proteins. After *trans*-Golgi, the cargo can be trafficked to three destinations: 1) the plasma membrane, 2) the secretory granules, and 3) the endosomes [44]. MT1-MMP is stored in endosomes upon endocytosis [45], [46], [47] and during exocytosis [48]. Our data suggest that both KIF3A and KIF13A initially traffic MT1-MMP vesicles in coordination from *trans*-Gogi to endosomes, and then KIF13A alone further transports the vesicles to the leading edge (Fig. 9). We found that KIF3A-Halo and KIF13A-GFP in part colocalized with MT1-RFP in the endosomes and the *trans*-Golgi (Fig. 7). Since it was observed that the velocity of MT1-RFP vesicles in the perinuclear region became faster when both KIF3A-Halo and KIF13A-GFP were ectopically co-expressed compared to when each KIF was co-expressed with MT1-RFP (Fig. 5 and 6), these KIFs indeed cooperate in vesicle transport between the *trans*-Golgi and endosomes. As the movie S7 indicated, KIF13A is solely responsible for transporting MT1-RFP vesicles laterally to the cell periphery as KIF3A stayed in the perinuclear area. However, the knockdown of KIF3A completely eliminated lateral movement of MT1-RFP vesicles (movies S9), suggesting that the vesicle transport from the *trans*-Golgi to the endosomes cannot be achieved without KIF3A. Therefore, we have concluded that coordination of these two KIFs between the *trans*-Golgi and the endosomes is essential for MT1-MMP vesicle transport to the cell periphery (Fig. 9). KIF3A belongs to the kinesin-2 subfamily and has been shown to play a crucial role in primary cilium formation [49]. It was demonstrated that KIF3 is involved in post-Golgi transport of N-cadherin/β-catenin, which plays a role in the adhesion of neuronal progenitor cells [30]. KIF13A belongs to the kinesin-3 subfamily and mediate vesicle transport from the endosomal recycling compartment to the plasma membrane [24,31,32]. It has been shown that KIF13A, together with Rab22A and BLOCK1/2, plays a central role in the biogenesis of recycling endosomes [50].

There are three splicing variants of KIF9, KIF9-v1, -v2, and -v3. KIF9-v2 and -v3 encode identical amino acid sequences, while KIF9-v1 is 65 amino acids shorter in the coiled-coil region. Upon KIF9 knockdown using smart pool siRNA, KIF9-v1 was selectively knocked down. Interestingly colocalization with MT1-MMP vesicles was only observed with KIF9-v1, but not KIF9-v2. Therefore, increased gelatin and collagen film degradation and proMMP-2 activation resulting from KIF9 knockdown were most likely attributed to KIF9-v1 knockdown, which is the first example of functional difference between KIF9 variants. However, it is unclear why KIF9-v1, but not KIF9-v2, is selectively involved in MT1-MMP vesicle transport since KIF9-v1 and v2 share the same C-terminal tail region, where these KIFs are thought to interact with the cargo. Further investigation is required.

Live-cell imaging data indicated that the vesicles positive for MT1-RFP and KIF9-v1-GFP did not move much in a lateral direction (Supplementary Fig S3). However, they faded out from the focus plane over time, suggesting that KIF9 may carry the vesicles to the dorsal side of the cell surface. Increased gelatin film degradation upon KIF9 knockdown was inhibited by co-silencing *kif13a* or *kif3a* genes, suggesting that the increased degradation is due to increased KIF13A- and KIF3A-dependent MT1-MMP vesicle transport to the matrix attachment sites (Supplementary Fig S4). It is thus possible that KIF9-v1 may antagonize with KIF13A and KIF3A to bring MT1-MMP-containing vesicles to different plasma membrane domains (Fig. 9). Upon KIF9-v1 knockdown, vesicle transport by KIF13A and KIF3A started to dominate, resulting in increased matrix degradation.

KIF9 knockdown increased cellular invasion in trans-well invasion assay, with the same tendency observed for gelatin and collagen film degradations. However, KIF9 knockdown significantly reduced cell migration in microcarrier beads invasion assay. This seemingly contradictory result can be due to differences between the two invasion assay systems. The first difference is whether cells face the collagen only at the ventral side (trans-well system) or surrounded by collagen fibrils (micro-carrier beads system). Another difference can be whether the migration is chemoattractant-guided or matrix-guided. In the trans-well system, the lower chamber contained 10% FBS, while the upper chamber was serum-free. Thus, cells in the upper chamber are attracted by the serum components such as lysophosphatidic acid [51] in the lower chamber. In the microcarrier beads invasion assay, there are no chemoattractant gradients. Thus, cells are instead guided by collagen fibrils. When cells migrate through the 3D collagen matrix, cells enlarge the gaps of collagen fibrils by degrading them at the middle of pseudopods in a ring shape [40,52]. KIF9 may be involved in the vesicle trafficking of MT1-MMP to these regions of the plasma membrane in coordination with KIF3A and KIF13A. KIF9 was previously shown to be involved in gelatin degradation at podosomes in macrophages [53]. In that report, they found that reggie/flotillin interacts with the C-terminal cargo binding domain of KIF9, and knockdown of KIF9 or flotillin significantly reduced gelatin degradation at the podosomes [53]. However, it was concluded that KIF9 was not directly involved in MT1-MMP vesicle transport to the podosomes in macrophages [22,53]. The detailed pathway of KIF9-dependent vesicle transport needs further investigation.

Besides cancer cells, human rheumatoid synovial cells (RASFs) also use MT1-MMP to achieve cartilage erosion in RA [28,[54], [55], [56]]. Our data indicate that knockdown of KIF3A and KIF13A in human RASFs significantly reduced collagen film degradation. In contrast, KIF9 knockdown enhanced it (Supplementary Fig S5), suggesting that RASFs share the same vesicle transport mechanism with HT1080 cells. It has been reported that KIF5B and KIF3A/KIF3B are the responsible KIFs in trafficking MT1-MMP vesicles to the podosome in macrophages [22]. KIF5B and KIF3A were also shown to mediate MT1-MMP vesicle transport to the invadopodia in MDA-MB231 [25]. However, our data indicate that knockdown of KIF5B did not influence gelatin film degradation in HT1080 cells, most likely due to the difference in MT1-MMP vesicle transport pathway between these cells. Macrophages deliver the MT1-MMP vesicles to the podosomes and MDA-MB231 cells to the invadopodia. HT1080 cells transport the vesicles to focal adhesion [20] and lamellipodia [57,58]. Thus, KIFs involved in MT1-MMP vesicles are likely to be cell type-dependent. Nevertheless, we have concluded that KIF5B is not involved in the vesicle transport of endogenous MT1-MMP in HT1080 cells.

The analysis of the TCGA database revealed that the expression profile of KIF13A and KIF9 is altered in several cancer types (Supplementary Figs S6a–S6c). Amplification is the most common *Kif13a* gene alteration detected across the TCGA database. KIF13A is reported by the Pathology Atlas (https://www.proteinatlas.org/humanproteome/pathology) [39] as an unfavorable prognostic marker for liver cancer patients. As shown in Supplementary Fig S6d, liver cancer patients with a high expression of KIF13A had a significantly lower survival probability than those with a low expression. Our data suggest that KIF13A is a crucial player of MT1-MMP intracellular trafficking pathways and mediate MT1-MMP-dependent invasion of cancer cells. Therefore, a pro-tumorigenic role of *Kif13a* may be its involvement in MT1-MMP trafficking to the cell surface. KIF9 is mainly deleted rather than amplified across the TCGA database. According to the Pathology Atlas, it is a favorable prognostic marker in renal and colorectal cancers (Supplementary Figs S6e and S6f). Thus, KIF9 may have an anti-tumorigenic potential for these two cancer types. These data highlight that KIF13A and KIF9 may have cancer type-specific roles, at least partly due to their roles in MT1-MMP intracellular trafficking. Chandrasekaran et al. [59] used cBioPortal to search for kinesin gene alterations in TCGA. They reported that the *Kif13a* gene was amplified in more than 10% of patients diagnosed with serous ovarian adenocarcinoma or urothelial bladder carcinoma [59]. They also found that 12% of patients with clear renal carcinoma had a homozygous deletion of the *Kif9* gene [59]. These values were slightly different from our analysis of the TCGA database. The discrepancies are likely due to the large number of data added to the database after publication. Cho *et al*. [60] performed an integrated analysis of specific kinesin's clinical significance, including KIF9, in low-grade glioma and glioblastoma. They showed that high KIF9 expression is linked to cancer progression and significantly lower survival probability, especially for patients diagnosed with glioblastoma [60]. Thus, the role of KIF9 may be cancer-specific.

In conclusion, we have identified KIF3A, KIF13A, and KIF9-v1 as the major KIFs that traffic MT1-MMP vesicles in HT1080 cells. KIF3A and KIF13A collaborate and transport MT1-MMP to the leading edge, while KIF9-v1 seems to work against KIF13A and KIF3A by trafficking MT1-MMP-containing vesicles to non-leading edge membrane structures. Our findings revealed novel mechanisms of interplay between different KIFs, which contribute to understanding vesicle transport mechanisms during cancer invasion.

## Materials and methods

### Plasmid constructs

MT1-RFP in pSG5 vector (Agilent, Cheshire, UK) was generated as described previously [43]. cDNAs encoding human KIF13A, KIF3A, KIF9-v1, KIF9-v2, and KIF1C were amplified by PCR using a cDNA library from HT-1080 cells as a template. The AcGFP was inserted at the N-terminus of each KIFs with three glycines as a linker. The mutants were constructed by the overlap extension PCR method. KIF13A-GFP (forward primer for AcGFP: 5’-TAGGAGCTCGGTACCGCCGCCACCATGGTGAGCAAGGGCG-3’; reverse primer: 5’-CCATTCCACCTCCCTTGTACAGCTATCCATGC-3’; forward chimera primer for AcGFP-KIF13A: 5’-TAGGAGCTCGGTACCGCCGCCACCATGGTGAGCGCAAGGGCG-3’; reverse flanking primer: 5’-TAGCCCGGGTCACTTGTACAGCTCATCCATGC-3’). KIF3A-GFP (forward flanking primer: 5’- ATACGACTCACTATAGGGCGAATTCGAGCCACCATGGTGAGCAAGGGCGCC-3’; reverse primer: 5’-CGGCATTCCACCTCCCTTGTACACTCATCCATGCCGTG-3’; forward chimera primer for AcGFP-KIF3A: 5’-TACAAGGGAGGTGGAATGCCGATCGGTAAATCAGA-3’; flanking reverse primer: 5’-CCTCTTCATCATCATCTTCC-3’), KIF9-v2 (forward flanking primer: 5’- AATTCGAGCTCGGTACCCAGATCTGCCACCATGGTGAGCAAGGGCG-3’; reverse primer: 5’-CCCATTCCACCTCCCTTGTACAGCTCATCCATGCCG-3’; forward chimera primer for AcGFP-KIF9-v2: 5’-ACAAGGGAGGTGGAATGGGTACTAGGAAAAAAGTTC-3’; flanking reverse primer: 5’-AATAAGATCTGGATCCCCCTATTTTCTATGTGCCTGCTG-3’) and KIF1C-GFP (forward flanking primer: 5’- AARRCGAGCTCGGTACCCGCCACCATGGTGAGCAAGGGCGCC-3’; reverse primer: 5’-GCCATTCCACCTCCCTTGTACAGCTCATCCATGCCGTG-3’; forward chimera primer for AcGFP-KIF1C: 5’-TACAAGGGAGGTGGAATGGCTGGTGCCTCGGTCAA-3’; flanking reverse primer: 5’-AATAAGATCTGGATCCCCTCACACAGCTGCCCCACTCTC-3’). KIF9-v1-GFP was generated by restriction enzyme cloning. KIF3A-HaloTag and KIF9-v2-HaloTag were generated by sub-cloning KIF3A and KIF9-v2 into pHTN HaloTag CMV-neo (Promega, Southampton, UK). KIF13A, KIF13A-GFP, KIF9-v1, KIF9-v2, KIF9-v1-GFP, KIF9-v2-GFP, KIF1C, and KIF1C-GFP were subcloned into pSG5 vector.

### Cell culture, transient transfection, and siRNA treatment

HT-1080 human fibrosarcoma cells (ECACC, Salisbury, UK) were cultured in Dulbecco's modified Eagle's medium (DMEM) (Lonza, Basel Switzerland), containing 10% FBS (Gibco, ThermoFisher, UK), penicillin/ streptomycin (P/S) (PAA). Rheumatoid arthritis synovial fibroblasts derived from three patients were cultured in DMEM supplemented with 20% FBS and P/S [54]. HT-1080 cells were transfected with plasmid constructs using Trans-IT2020 (Mirus Bio, Madison WI, USA) according to the manufacturer's instructions. Gene silencing was performed by transfection of SMARTpool ON-TARGETplus siRNA (Dharmacon, Thermo Fisher, Waltham, US) using INTERFERin (Polyplus-transfection, New York, NY, USA) according to the manufacturer's instructions. Non-targeting siRNA (NT-siRNA) was purchased from Dharmacon (ThermoFisher). Gene silencing effectiveness was tested by Western blotting (WB) or RT-PCR. Cells were subjected to the experiments after 72 h of transfection.

### SDS-PAGE and Western blotting

Cell lysates were prepared by directly dissolving in 1xSDS loading buffer containing 0.1% 2-mercaptoethanol. Cell lysates were subjected to SDS-PAGE, and the proteins in the gel were transferred to a PBDF membrane using Trans-Blot Turbo Transfer System (Bio-Rad, Watford, UK). After probing the membrane with the primary antibodies, the bands were visualized using fluorescently-labeled secondary antibodies (LI-COR, Nebraska, USA). Membranes were scanned by the Odyssey CLx imaging system (LI-COR). The band intensities were quantified by ImageJ software (National Institutes of Health). Actin or tubulin bands were used for normalization. Mean band intensities were plotted using Prism (GraphPad Software, Inc., San Diego, CA, USA). Statistical significance was calculated using either a parametric unpaired T-test or a one-way ordinary ANOVA with Tuckey's multiple comparison test. The following primary antibodies were used:

Rabbit anti-MT1-MMP monoclonal antibody (clone EP1264Y, ab51074, Abcam, Cambridge, UK), mouse anti-MT1-MMP monoclonal antibody (clone 222-1D8), rabbit anti-KIF1C polyclonal antibody (ab72238, Abcam), rabbit anti-KIF3A polyclonal antibody (ab11259, Abcam), mouse anti-actin monoclonal antibody (clone C4, sc47778, Santa Cruz, CA, USA), mouse anti-tubulin antibody (clone B-7, sc5286, Santa Cruz). The following secondary antibodies were used: IRDye 680RD goat anti-mouse IgG (H + L) (926-68070, LI-COR), IRDye 800CW goat anti-rabbit IgG (H + L) (926-32211, LI-COR).

### RT-PCR

Total RNA was isolated using the RNAqueous Micro kit (Invitrogen, Thermo Fisher) according to the manufacturer's instructions. RNA was reversed-transcribed using the High Capacity cDNA Reverse Transcription kit from AB applied Biosciences (Thermo Fisher). Resulted cDNA was used as a template for PCR using the Dream Taq Polymerase (Thermo Fisher). GADPH was used as a housekeeping gene. The following primers were used: KIF13A (forward: 5’-TTTCCAGTAGGAGGAGTC-3’; reverse: 5’-AAGTTGTTGCGGTGAAGG-3’), KIF3A (forward: 5’-TGCAAAGTCAGAGATGGC-3’; reverse: AGCTGCCATTCTCCTATG-3’), KIF9 (forward: 5’-CCCGGACCTTATCAGAGGAAAAG-3’; reverse (v1): 5’-GGTGTCGGGCCTGAGTGG-3’; reverse (v2): 5’-GGATGGGACAAGCTGGGTC-3’), KIF1C (forward: 5’-TTCCAGCCCAAAAAGCAC-3’; reverse: 5’-CGGACCTTCTCTCTCATC-3’), KIF5B (forward: 5’-GCTACAAGAGTTAAAAAGAGTGCT-3’; reverse: 5’-TCACACTTGTTTGCCTCCTCCAG-3’), MT1-MMP (forward: 5’- GGGACCTACGTACCCACACA; reverse: 5’-TAGCGCTTCCTTCGAACATT-3’) AND GADPH (forward: 5’-TTCACCACCATGGAGAAGGC-3’; reverse: 5’- GGTCCCTCCGATGCCTGC-3’). RT-PCR gels were quantified by Fiji using the same protocol described above to quantify WB bands. GADPH bands were used for normalization.

### Gelatin film degradation assay

Fluorescently-labeled gelatin-coated coverslips were prepared as described previously [61,62]. Cells were cultured atop of the gelatin-coated coverslips for 2-15 h, depending on the assay, in the presence or absence of GM6001 (10 μM) (Elastin Products Company, Missouri, USA), TIMP-1 (200 nM), or DX-2400 (200 nM). TIMP-1 was a gift from Prof Gillian Murphy (University of Cambridge), and DX-2400 was a gift from Dyax Corp. Cells were then fixed in 4% formaldehyde (Sigma Aldrich, Merck Life Science UK, Ltd., Dorset, UK) in PBS for 15 min and stained with DAPI (Sigma-Aldrich). Images were acquired with a Nikon microscope using the 10X dry lens (NA = 0.3) on a Nikon TE2000-E microscope equipped with an ORCA-ER CCD camera (Hamamatsu Photonics, Shizuoka, Japan) operated by Volocity Acquisition module software (Improvision). Gelatin film degradation was quantified using Fiji. The degradation area was calculated using the gelatin fluorescence image, which was converted to greyscale and thresholded. The threshold was set the same for all the pictures analyzed, to have an objective mean of analysis. Area and area fractions were measured. The DAPI-stained corresponding image was converted to a binary image to count the number of cells in the microscopic field. The degraded area per cell was calculated as follow:

$$Degraded\;Area\;per\;cell\;\left({\mu m}^{2}\right)=\;\frac{Total\;Degraded\;Area}{Number\;of\;Cells}$$

Mean degraded areas were plotted using Prism, and statistical significance was calculated using one-way ordinary ANOVA with Tuckey's multiple comparison test.

### Collagen film degradation assay

Collagen film degradation was carried out as described previously [61,62]. Briefly, PureCol (bovine collagen type-I, Advanced Biomatrix, San Diego, CA, USA) and Cellmatrix type I-A collagen (Nitta Gelatin, Osaka, Japan) were mixed in the ratio of 1:1. This mixture was neutralized and diluted to 2 mg/ml. 12-well multi-well plates were coated with 100 μl collagen solution/well and set for gelation. Cells were cultured on the collagen film for 72 h in the presence or absence of GM6001 (10 μM), TIMP-1 (200 nM), or DX-2400 (200 nM). Cells were removed extensively by trypsin/EDTA (Lonza), and the remaining collagen layer was fixed with 4% formaldehyde in PBS and stained with R-250 Coomassie Brilliant Blue (Thermo Fisher). Representative images were acquired using the 10X dry lens (NA = 0.3) on the Nikon TE2000-E microscope equipped with an ORCA-ER CCD camera (Hamamatsu Photonics) operated by Volocity Acquisition module software (Improvision, PerkinElmer). Collagen degradation was quantified using Fiji by measuring the integrated density of the collagen layer. Mean results were plotted using Prism, and statistical significance was calculated using one-way ordinary ANOVA with Tuckey's multiple comparison test.

### Zymography

Gelatin zymography was conducted as reported previously [61,62]. Enzyme activity was visualized directly on the gels as negative staining bands with Coomassie Blue. Pro-MMP-2 (P), intermediate MMP-2 (I), and active MMP-2 (A) bands were quantified using Fiji. The percentage of processed pro-MMP-2 over the total was calculated as follow:

$$\frac{Area\;Peaks\;\left(I+A\right)}{Area\;Peaks(\;P+I+A)}*100$$

### Cell Surface biotinylation

Surface biotinylation was carried out using Sulfo-NHS-biotin (Thermo Fisher) as described previously [41]. Cells were cultured to confluency, and cell surface proteins were labeled with sulfo-NHS-biotin (Thermo-Fischer), followed by affinity precipitation of biotinylated molecules by streptavidin-conjugated Dyna beads (Thermo Fisher). The eluted samples were subjected to Western Blotting analyses using rabbit monoclonal anti-MT1-MMP antibody.

### Indirect immunofluorescent staining

Cells cultured atop of un-labelled or fluorescently labelled gelatin were fixed with 4% (v/v) formaldehyde in PBS for 5 min and blocked with 3% (w/v) BSA, 5% (v/v) goat serum in TBS (blocking solution) for 1 h at room temperature (RT). Cells were then incubated with primary antibodies in a blocking solution. The cells were further probed by Alexa488-conjugated goat anti-mouse IgG (Molecular Probes), DyLight650-conjugated goat anti-rabbit IgG (Thermo Fisher), Alexa568-conjugated Phalloidin (Molecular Probes, Thermo Fisher), or DAPI. The following primary antibodies were used for staining: rabbit anti-MT1-MMP monoclonal antibody (clone EP1264Y, Abcam), mouse anti-human β1 integrin monoclonal antibody (clone 12G10, Millipore). Cell surface staining for MT1-MMP and β1 integrin was carried out by staining non-permeabilized cells. Under the condition, antibodies only recognize cell surface molecules.

### Trans-well invasion assay

Trans-well invasion assay was performed as previously described [63]. Briefly, a 24-well insert with an 8-μm-pore membrane Trans-wells (VWR International Ltd, Radner, PA, USA) was coated with 50 μl of CellMatrix/PureCol collagen mixture (1:1, 2 mg/ml), incubated at 37°C for 1 h to set the collagen and dried overnight at RT. Cells (2 × 10^4^/well) were seeded in the upper chamber and cultured for 18 h. Lower chamber media contained 10% FBS, while upper chamber media was serum-free. Invaded cells were stained with DAPI, imaged with fluorescence microscopy, and analyzed by Volocity software.

### Microcarrier beads invasion assay

Microcarrier beads invasion assay was carried out as described previously [63]. Cells were attached to gelatin-coated Cytodex 3 microcarrier beads (VWR) by preparing a cell/bead suspension incubated on a shaker for 6 h at 37°C. Beads coated with cells were suspended in neutralized Cellmatrix collagen (final concentration 2 mg/ml) and incubated overnight in the presence or absence of GM6001 (10 μM). The invasion was analyzed as the distance between a cell nucleus and the bead's surface using the line tool of ImageJ. Migrated distances were calculated for fifty cells per treatment. Mean migrated distances were plotted using Prism, and statistical significance was calculated using one-way ordinary ANOVA with Tuckey's multiple comparison test.

### Wound-healing assay

Ibidi Culture-Inserts (Ibidi, Gräfelfing, Germany) consisting of two reservoirs separated by a 500-µm-width wall was placed in each well of a 24-well plate, and six inserts were used for each treatment. Cells were seeded in the two reservoirs of the inserts and incubated until confluence. After the inserts were gently removed, cells were further cultured for 6 h. Pictures were taken immediately after the inserts were removed and at the 6 h time point. To measure the percentage of wound closure, ImageJ was employed. The ROI corresponding to the initial wound-gap (ROI-I) and the one delimited by the migration front after 6 h of incubation (ROI-F) were calculated for each condition. The percentage of wound closure was calculated as follow:

$$Wound\;Closure\;\left(\%\right)=\;\frac{\left(ROI-I\right)-(ROI-F)}{ROI-I}*100$$

Mean percentages were plotted using Prism, and statistical significance was calculated using one-way ordinary ANOVA with Tuckey's multiple comparison test.

### Live cell imaging

Cells were transfected with GFP-tagged KIFs, Halo-tagged KIFs, and MT1-RFP. After 36 h, they were seeded at a density of 4 × 10^3^/well on gelatin-coated glass-bottomed 8-well chambers (Ibidi). After 12 h, live images were acquired by either confocal laser scanning microscopy or total internal reflection fluorescence (TIRF) microscopy (details specified below). Both microscopes were equipped with an environmental chamber to maintain the temperature at 37°C and CO_2_ at 5%. Depending on the experiment, time-lapse images were acquired every 2 to 30 sec.

### Image acquisition

All widefield images were captured on an inverted Nikon TE2000-E widefield microscope with Volocity Acquisition software (PerkinElmer, Coventry, UK). These objective lenses were used: 4X objective lens (Plan Fluor 4X/NA 0.13), 10X objective lens (UPLSAPO 10X/NA 0.30 DIC), and 20X objective lens (UPLSAPO 20X/NA 0.45). Confocal laser scanning microscopy imaging was performed on a PerkinElmer Spinning Disk Confocal Microscope based on a Nikon TE 2000-U Eclipse motorized inverted microscope with DIC optics. A 60X objective lens was used: 60X (Plan Apo 60 × /NA 1.40). Volocity software (PerkinElmer, Coventry, UK) was used for Acquisition. Some images and movies were also acquired using Zeiss LSM980 with Airyscan 2 with 63x objective lens (C Plan-Apochromat 63x/1.4 Oil DIC M27) in the Super resolution mode for the still imaging (Fig. 7 and 8) and confocal mode for the live cell imaging (movies S8-S11). For TIRFM, an Olympus microscope in TIRF mode (CellTIRF-4Line system; Olympus), equipped with an EMCCD camera (Evolve), was used. A 150X objective lens was used (UPLSAPO 150 X/ NA 1.45).

### Data analysis

To measure the amount of cell surface β1 integrin and MT1-MMP at the substrate-attachment side, the corresponding fluorescence intensities (FIs) were measured by ImageJ. TIRF images were analyzed by defining a region of interest (ROI), corresponding to the cell body (ROI-CB), and a rectangular ROI in the background (ROI-B). FIs were calculated as follow:

FI= FI(ROI-CB)-FI(ROI-B)

30 cells were analyzed for each condition. ROI-B dimension and position were kept the same throughout the analysis. Mean FIs were plotted using Prism, and statistical significance was calculated using one-way ordinary ANOVA with Tuckey's multiple comparison test.

To measure the overall amount of MT1-MMP on the cell surface of HT-1080 cells, FIs were analyzed by Volocity measurement module software. Extended focus images of HT-1080 cells stained for cell surface endogenous MT1-MMP, captured by spinning disk confocal microscope, were used. Thirty cells were analyzed for each condition. Mean FIs were plotted using Prism, and statistical significance was calculated using one-way ordinary ANOVA with Tuckey's multiple comparison test.

Co-localisation between GFP-tagged KIFs and MT1-RFP was quantified using the Coloc 2 plugin in Fiji, which calculates Pearson's correlation coefficients (PCCs). This procedure was used on two-color channel images of cells cultured in 2D matrices and two-color channel stacks of cells cultured in 3D collagen matrices. Mean PCCs were plotted using Prism, and statistical significance was calculated using one-way ordinary ANOVA with Tuckey's multiple comparison test.

Tracking of MT1-RFP-containing vesicles trafficked by GFP-tagged KIFs was performed using the Track Mate plugin [64] in Fiji. Vesicles were identified using the LoG detector, and their diameter was estimated at 1.5 μm. Tracking was performed by Simple LAP Tracker using 2 μm as linking and gap-closing maximum distances. The gap-closing maximum frame gap was set at 2. Mean velocities and vesicle diameters were calculated automatically by the plugin. Data were plotted using Prism, and statistical significance was calculated by a parametric unpaired T-test or a one-way ordinary ANOVA with Tuckey's multiple comparison test.

To analyze genomic alterations of Kif13a, Kif3a, and Kif9 genes in cancer, we employed cBioPortal [37,38] (https://www.cbioportal.org). The Cancer Genome Atlas (TCGA) PanCancer Atlas studies were selected to visualize and analyze Kif13a, Kif3a, and Kif9 gene alterations. The Human Protein Atlas (https://www.proteinatlas.org) was used to check whether Kif13a Kif3a or Kif9 genes were prognostic markers for specific cancer types.

## Appendix Supplementary materials

Image, application 1

Image, application 2

Image, application 3

Image, video 4

Image, video 5

Image, video 6

Image, video 7

Image, video 8

Image, video 9

Image, video 10

Image, video 11

Image, video 12

Image, video 13

Image, video 14
