# Supplementary material for: Coordination of two kinesin superfamily motor proteins, KIF3A and KIF13A, is essential for pericellular matrix degradation by membrane-type 1 matrix metalloproteinase (MT1-MMP) in cancer cells
Source: Matrix Biol. 2022 Mar;107:1–23. doi: 10.1016/j.matbio.2022.01.004 (PMC9355896; doi:10.1016/j.matbio.2022.01.004)
Supplement: Supplementary file 1 [file mmc1.pdf]

**Table S1. Excluded KIFs from screening**
There are 45 KIFs genes in human. Based on the literatures, 28 KIFs including splicing variant were excluded from the screening. The list of excluded KIFs and the reason with the references were shown.

| Excluded KIFs | | |
| --- | --- | --- |
| Kinesin # | KIF # | Reason for being excluded with references |
| Kinesin-1 | KIF5A<br>KIF5C | Neuron specific [1] |
| Kinesin-2 | KIF17 | Neuron specific [2] [3] |
| Kinesin-3 | KIF1A<br>KIF14<br>KIF16A<br>KIF16B<br>KIF28 | Neuron specific [4]; Mitotic kinesins [5]; or function unknown |
| Kinesin-4 | KIF4A<br>KIF4B | Chromokinesins, expressed mostly in the juvenile brain [6, 7]. |
| Kinesin-5 | KIF11 | Mitotic kinesin [8, 9] |
| Kinesin-6 | KIF20A<br>KIF20B<br>KIF23 (v1,v2) | Mitotic kinesin[10]. |
| Kinesin-7 | KIF10 | Mitotic kinesin[11] |
| Kinesin-8 | KIF19A<br>KIF19B<br>KIF18A<br>KIF18B | Unknown function[12, 13]; Mitotic kinesin[14] |
| Kinesin-9 | KIF6 | Testis-specific[15] |
| Kinesin-10 | KIF22 | Chromokinesin[16] |
| Kinesin-11 | KIF26A<br>KIF26B | Microtubule-independent[17] |
| Kinesin-12 | KIF12 | Mitotic kinesin[18]. |
| Kinesin-13 | KIF2A<br>KIF2B<br>KIF2C | M-type kinesin with microtubule-depolymerising activity[19] |
| Kinesin-14 | KIFC1<br>KIFC2<br>KIFC3<br>KIF25 (v1,v2) | C-type kinesin which moves towards the minus end of microtubule; implicated in acrosome formation [20] [21]. |

## REFERENCES

[1] Y. Kanai, Y. Okada, Y. Tanaka, A. Harada, S. Terada, N. Hirokawa, KIF5C, a novel neuronal kinesin enriched in motor neurons, J Neurosci 20(17) (2000) 6374-84.

[2] L. Guillaud, M. Setou, N. Hirokawa, KIF17 dynamics and regulation of NR2B trafficking in hippocampal neurons, J Neurosci 23(1) (2003) 131-40.

[3] M. Setou, T. Nakagawa, D.H. Seog, N. Hirokawa, Kinesin superfamily motor protein KIF17 and mLin-10 in NMDA receptor-containing vesicle transport, Science 288(5472) (2000) 1796-802.

[4] Y. Okada, H. Yamazaki, Y. Sekine-Aizawa, N. Hirokawa, The neuron-specific kinesin superfamily protein KIF1A is a unique monomeric motor for anterograde axonal transport of synaptic vesicle precursors, Cell 81(5) (1995) 769-80.

[5] U. Gruneberg, R. Neef, X. Li, E.H. Chan, R.B. Chalamalasetty, E.A. Nigg, F.A. Barr, KIF14 and citron kinase act together to promote efficient cytokinesis, J Cell Biol 172(3) (2006) 363-72.

[6] Y. Sekine, Y. Okada, Y. Noda, S. Kondo, H. Aizawa, R. Takemura, N. Hirokawa, A novel microtubule-based motor protein (KIF4) for organelle transports, whose expression is regulated developmentally, J Cell Biol 127(1) (1994) 187-201.

[7] Y. Kurasawa, W.C. Earnshaw, Y. Mochizuki, N. Dohmae, K. Todokoro, Essential roles of KIF4 and its binding partner PRC1 in organized central spindle midzone formation, EMBO J 23(16) (2004) 3237-48.

[8] K.E. Sawin, T.J. Mitchison, L.G. Wordeman, Evidence for kinesin-related proteins in the mitotic apparatus using peptide antibodies, J Cell Sci 101 ( Pt 2) (1992) 303-13.

[9] L.C. Kapitein, E.J. Peterman, B.H. Kwok, J.H. Kim, T.M. Kapoor, C.F. Schmidt, The bipolar mitotic kinesin Eg5 moves on both microtubules that it crosslinks, Nature 435(7038) (2005) 114-8.

[10] R.D. Fontijn, B. Goud, A. Echard, F. Jollivet, J. van Marle, H. Pannekoek, A.J. Horrevoets, The human kinesin-like protein RB6K is under tight cell cycle control and is essential for cytokinesis, Mol Cell Biol 21(8) (2001) 2944-55.

[11] L. Ma, X. Zhao, X. Zhu, Mitosin/CENP-F in mitosis, transcriptional control, and differentiation, J Biomed Sci 13(2) (2006) 205-13.

[12] H. Miki, M. Setou, K. Kaneshiro, N. Hirokawa, All kinesin superfamily protein, KIF, genes in mouse and human, Proc Natl Acad Sci U S A 98(13) (2001) 7004-11.

[13] J. Stumpff, G. von Dassow, M. Wagenbach, C. Asbury, L. Wordeman, The kinesin-8 motor Kif18A suppresses kinetochore movements to control mitotic chromosome alignment, Dev Cell 14(2) (2008) 252-62.

[14] M.I. Mayr, S. Hummer, J. Bormann, T. Gruner, S. Adio, G. Woehlke, T.U. Mayer, The human kinesin Kif18A is a motile microtubule depolymerase essential for chromosome congression, Curr Biol 17(6) (2007) 488-98.

[15] T. Nakagawa, Y. Tanaka, E. Matsuoka, S. Kondo, Y. Okada, Y. Noda, Y. Kanai, N. Hirokawa, Identification and classification of 16 new kinesin superfamily (KIF) proteins in mouse genome, Proc Natl Acad Sci U S A 94(18) (1997) 9654-9.

[16] M. Ohsugi, K. Adachi, R. Horai, S. Kakuta, K. Sudo, H. Kotaki, N. Tokai-Nishizumi, H. Sagara, Y. Iwakura, T. Yamamoto, Kid-mediated chromosome compaction ensures proper nuclear envelope formation, Cell 132(5) (2008) 771-82.

[17] S.H. Lillie, S.S. Brown, Smy1p, a kinesin-related protein that does not require microtubules, J Cell Biol 140(4) (1998) 873-83.

[18] G.S. Lakshmikanth, H.M. Warrick, J.A. Spudich, A mitotic kinesin-like protein required for normal karyokinesis, myosin localization to the furrow, and cytokinesis in Dictyostelium, Proc Natl Acad Sci U S A 101(47) (2004) 16519-24.

[19] N. Homma, Y. Takei, Y. Tanaka, T. Nakata, S. Terada, M. Kikkawa, Y. Noda, N. Hirokawa, Kinesin superfamily protein 2A (KIF2A) functions in suppression of collateral branch extension, Cell 114(2) (2003) 229-39.

[20] W.X. Yang, A.O. Sperry, C-terminal kinesin motor KIFC1 participates in acrosome biogenesis and vesicle transport, Biol Reprod 69(5) (2003) 1719-29.

[21] W.X. Yang, H. Jefferson, A.O. Sperry, The molecular motor KIFC1 associates with a complex containing nucleoporin NUP62 that is regulated during development and by the small GTPase RAN, Biol Reprod 74(4) (2006) 684-90.
