# Supplementary material for: Coordination of two kinesin superfamily motor proteins, KIF3A and KIF13A, is essential for pericellular matrix degradation by membrane-type 1 matrix metalloproteinase (MT1-MMP) in cancer cells
Source: Matrix Biol. 2022 Mar;107:1–23. doi: 10.1016/j.matbio.2022.01.004 (PMC9355896; doi:10.1016/j.matbio.2022.01.004)
Supplement: Supplementary file 2 [file mmc2.docx]

**Supplemental movie legends**

**Movie 1. Live cell confocal imaging of HT1080 cells expressing MT1-RFP and KIF13A-GFP.**

HT1080 cells were transfected with expression plasmids for MT1-RFP and KIF13A-GFP. Cells were seeded on gelatin film coated coverslip-bottomed chamber and subjected to live-cell confocal imaging. Images were taken in every 3 seconds.

**Movie 2. Live cell confocal imaging of HT1080 cells expressing MT1-RFP and KIF13A-GFP.**

HT1080 cells were transfected with expression plasmids for MT1-RFP and KIF13A-GFP. Cells were seeded on gelatin film coated coverslip-bottomed chamber and subjected to live-cell confocal imaging. Images were taken in every 3 seconds.

**Movie 3. Live cell TIRF imaging of HT1080 cells expressing MT1-RFP and KIF13A-GFP.** HT1080 cells were transfected with expression plasmids for MT1-RFP and KIF13A-GFP. Cells were seeded on the gelatin film coated coverslip-bottomed chamber and subjected to live-cell TIRF imaging. Fluorescent and IRM images were taken in every 3 seconds.

**Movie 4. Live cell TIRF imaging of HT1080 cells expressing MT1-RFP and KIF13A-GFP.** HT1080 cells were transfected with expression plasmids for MT1-RFP and KIF13A-GFP. Cells were seeded on the gelatin film coated coverslip-bottomed chamber and subjected to live-cell TIRF imaging. Fluorescent and IRM images were taken in every 3 seconds.

**Movie 5. Live cell confocal imaging of HT1080 cells expressing MT1-RFP and KIF3A-GFP.**

HT1080 cells were transfected with expression plasmids for MT1-RFP and KIF3A-GFP. Cells were seeded on gelatin film coated coverslip-bottomed chamber and subjected to live-cell confocal imaging. I mages were taken in every 3 seconds.

**Movie 6. Live cell confocal imaging of HT1080 cells expressing MT1-RFP and KIF3A-GFP.**

HT1080 cells were transfected with expression plasmids for MT1-RFP and KIF3A-GFP. Cells were seeded on gelatin film coated coverslip-bottomed chamber and subjected to live-cell confocal imaging. Images were taken in every 3 seconds.

**Movie 7. Live cell confocal imaging of HT1080 cells expressing MT1-RFP, KIF3A-HaloTag, and KIF3A-GFP.**

HT1080 cells were transfected with expression plasmids for MT1-RFP, KIF3A-HaloTag, and KIF3A-GFP. Cells were seeded on gelatin film coated coverslip-bottomed chamber and subjected to live-cell confocal imaging. KIF3A-HaloTag was reacted with Halo-ligand Coumarin for 15 min followed by brief wash of the cells prior to imaging. Images were taken in every 3 seconds.

**Movie 8. Live cell imaging of HT1080 cells expressing MT1-RFP and transfected with NT-siRNA.**

HT1080 cells were transfected with NT-siRNA followed by MT1-RFP plasmid. Cells were seeded on gelatin film coated coverslip-bottomed chamber and subjected to live-cell confocal imaging using Zeiss980. Images were taken in every 2 seconds.

Movie 9. Live cell imaging of HT1080 cells expressing MT1-RFP and transfected with KIF3A-siRNA. HT1080 cells were transfected with KIF3A-siRNA followed by MT1-RFP plasmid. Cells were seeded on gelatin film coated coverslip-bottomed chamber and subjected to live-cell confocal imaging using Zeiss980. Images were taken in every 2 seconds.

**Movie 10. Live cell imaging of HT1080 cells expressing MT1-RFP and transfected with KIF13A-siRNA.**

HT1080 cells were transfected with KIF13A-siRNA followed by MT1-RFP plasmid. Cells were seeded on gelatin film coated coverslip-bottomed chamber and subjected to live-cell confocal imaging using Zeiss980. Images were taken in every 2 seconds.

**Movie 11. Live cell imaging of HT1080 cells expressing MT1-RFP and transfected with KIF9-siRNA.**

HT1080 cells were transfected with KIF9-siRNA followed by MT1-RFP plasmid. Cells were seeded on gelatin film coated coverslip-bottomed chamber and subjected to live-cell confocal imaging using Zeiss980. Images were taken in every 2 seconds.
