# Supplementary material for: Coordination of two kinesin superfamily motor proteins, KIF3A and KIF13A, is essential for pericellular matrix degradation by membrane-type 1 matrix metalloproteinase (MT1-MMP) in cancer cells
Source: Matrix Biol. 2022 Mar;107:1–23. doi: 10.1016/j.matbio.2022.01.004 (PMC9355896; doi:10.1016/j.matbio.2022.01.004)
Supplement: Supplementary file 3 [file mmc3.pdf]

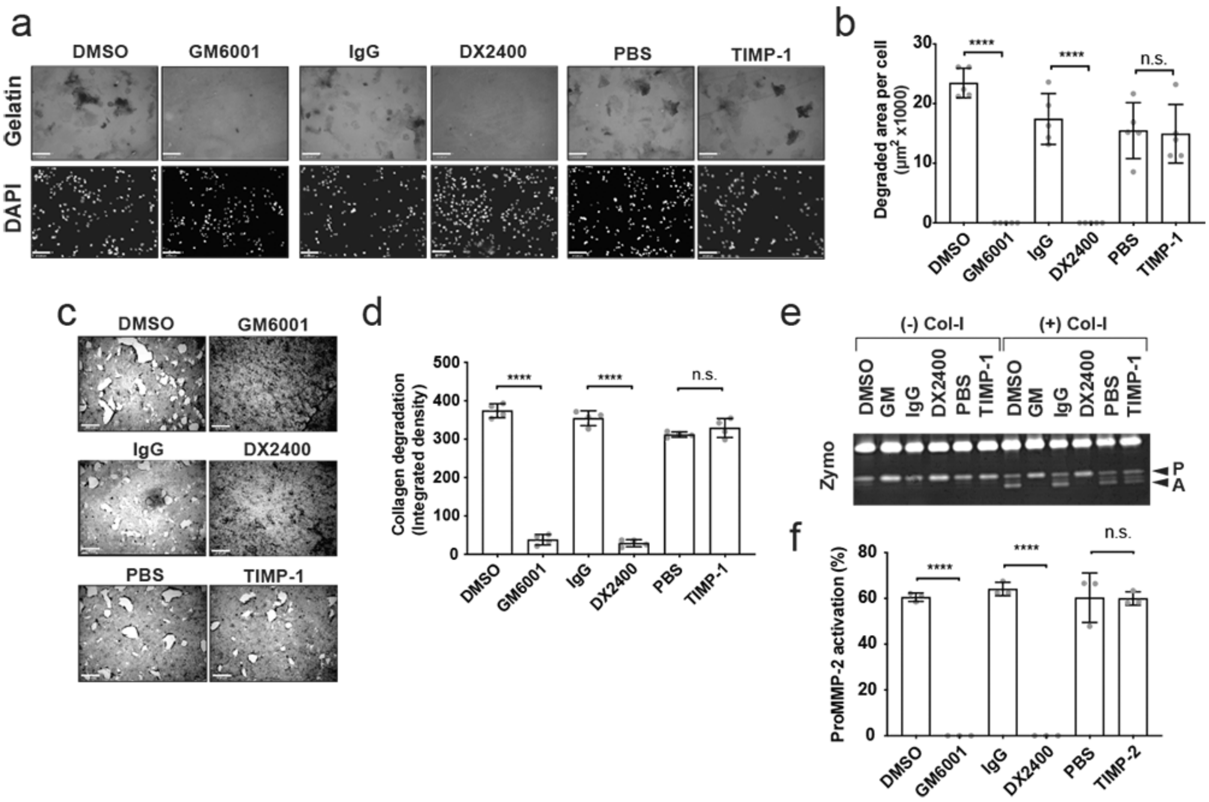

Figure S1. Gelatin and collagen film degradation and proMMP-2 activation are attributed to endogenous MT1-MMP in HT1080 cells.

a. HT-1080 cells were subjected to gelatin film degradation assay in the presence of GM6001 (10 μM), DX2400 (200 nM), TIMP-1 (200 nM) and the each control (DMSO, IgG, and PBS). Scale bars, 130 μm.

b. Quantification of the degradation area (μm^2) per cell in HT-1080 cells. Data are presented as mean ± SD of five different field of the images (n=5). Data are representative of three independent experiments. *P* value was calculated by One-way ordinary Anova with Tuckey's multiple comparisons test. ****$P < 0.0001$. n.s., non significant.

c. HT-1080 cells were subjected to collagen film degradation assay in the presence of GM6001 (10 μM), DX2400 (200 nM), TIMP-1 (200 nM) and the relative controls. Scale bars are 300 μm.

d. Quantification of the integrated density of the collagen layer in HT-1080 cells. Data are shown as mean ± SD (n=4) and are representative of three indipendent experiments. *P* value was calculated by One-way ordinary Anova with Tuckey's multiple comparisons test. ****$P < 0.0001$, n.s., non significant.

e. HT-1080 cells were cultivated in the presence or absence of collagen I (100 μg/ml) with the indicated inhibitors for 24 h. Culture media were analysed by zymography (Zymo). P, pro-MMP-2; A, active MMP-2.

f. Quantification of the percentage of MMP-2 processed forms (active and intermediate forms) over the total MMP-2 (proforms, intermediate, and active forms) upon collagen stimulation. Data are presented as mean ± SD (n=3) and the data shown is a representative of three indipendent experiments. *P* value was calculated by One-way ordinary Anova with Tuckey's multiple comparisons test. ****$P < 0.0001$, n.s., non significant.

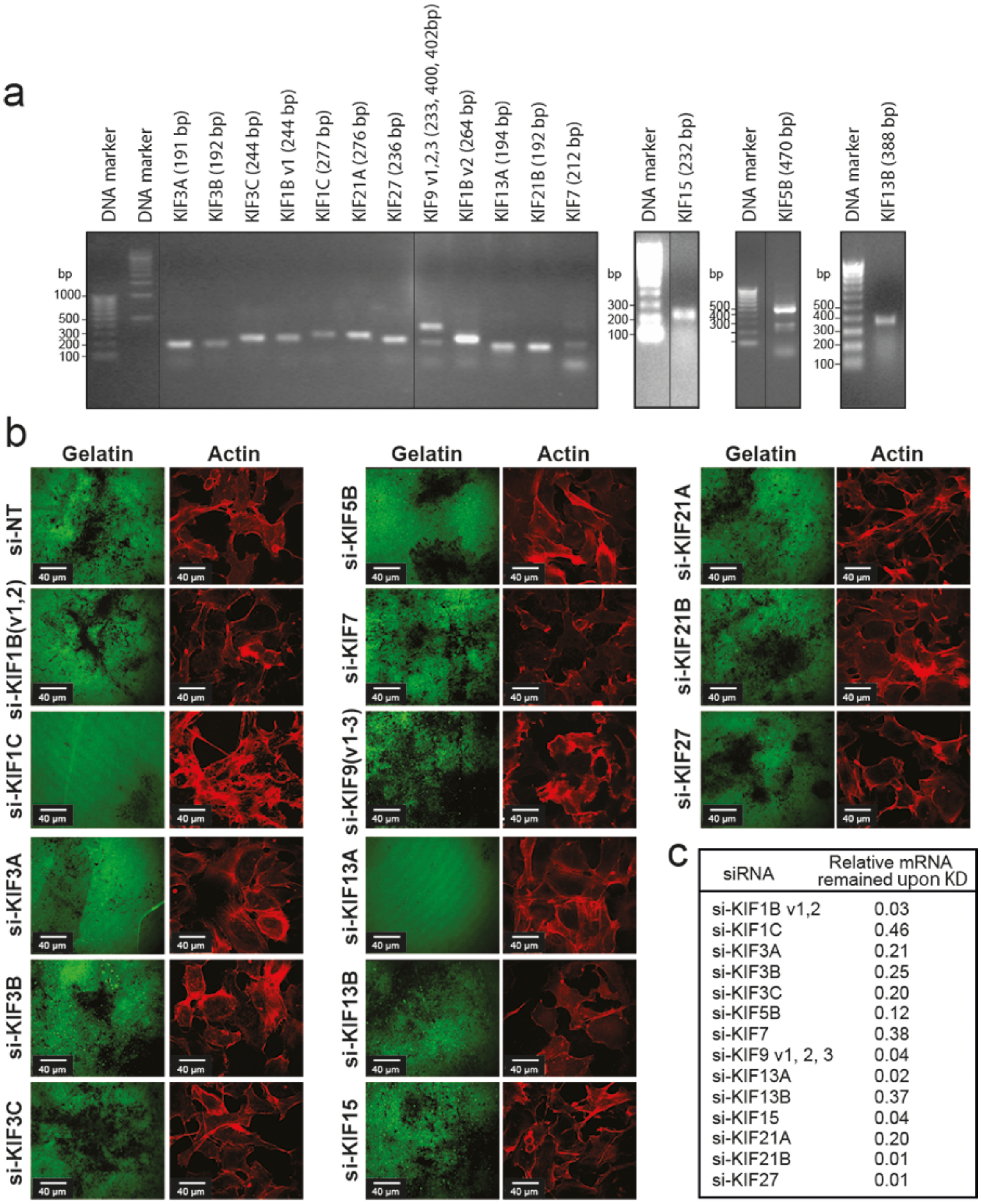

c

siRNA	Relative mRNA remained upon KD
si-KIF1B v1,2	0.03
si-KIF1C	0.46
si-KIF3A	0.21
si-KIF3B	0.25
si-KIF3C	0.20
si-KIF5B	0.12
si-KIF7	0.38
si-KIF9 v1, 2, 3	0.04
si-KIF13A	0.02
si-KIF13B	0.37
si-KIF15	0.04
si-KIF21A	0.20
si-KIF21B	0.01
si-KIF27	0.01

Figure S2. Screening KIFs involved in MT1-MMP vesicle trafficking for degradation of ECM substratum.

a. Expression of KIF genes screened in HT-1080. The mRNA extracted from HT1080 was subjected to RT-PCR (30 cycles) for expression of KIF genes selected for screening.

b. HT-1080 cells were transfected with siRNA for selected KIFs and subjected to Alexa488-gelatin film degradation assay.

c. Relative mRNA remained upon knockdown (KD) compared to non-target siRNA (si-NT)-transfected cells.

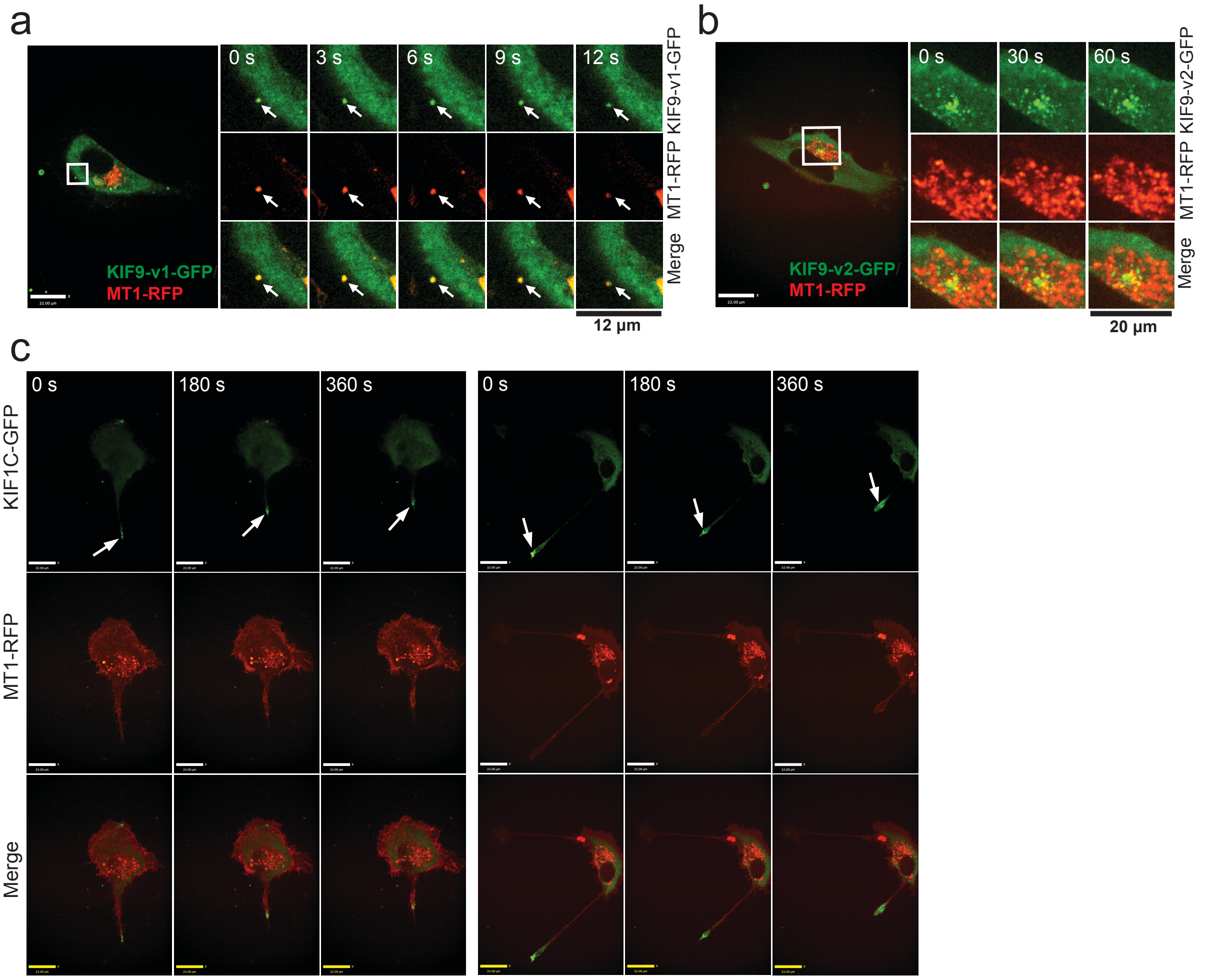

Figure S3. Time-lapse imaging of cells expressing MT1-RFP, KIF9-v1-GFP, KIF9-v2-GFP, and KIF1C-GFP.

a. HT-1080 cells transfected with KIF9-v1-GFP (green) and MT1-RFP (red) on gelatin film were subjected to live-cell imaging by confocal microscopy. A representative image sequence is shown. White arrows point vesicles of interest. The scale bar is 22 µm.

b. HT-1080 cells were transfected with KIF9-v2-GFP (green) and MT1-RFP (red), and they were subjected to live-cell imaging on gelatin film by confocal microscopy. A representative image time sequence is shown. Scale bars are 22 µm.

c. HT-1080 cells were transfected with KIF1C-GFP (green) and MT1-RFP (red), and they were subjected to live-cell imaging on the gelatin film by confocal microscopy. Representative image time sequences are shown. White arrows point trailing edges. Scale bars are 22 µm.

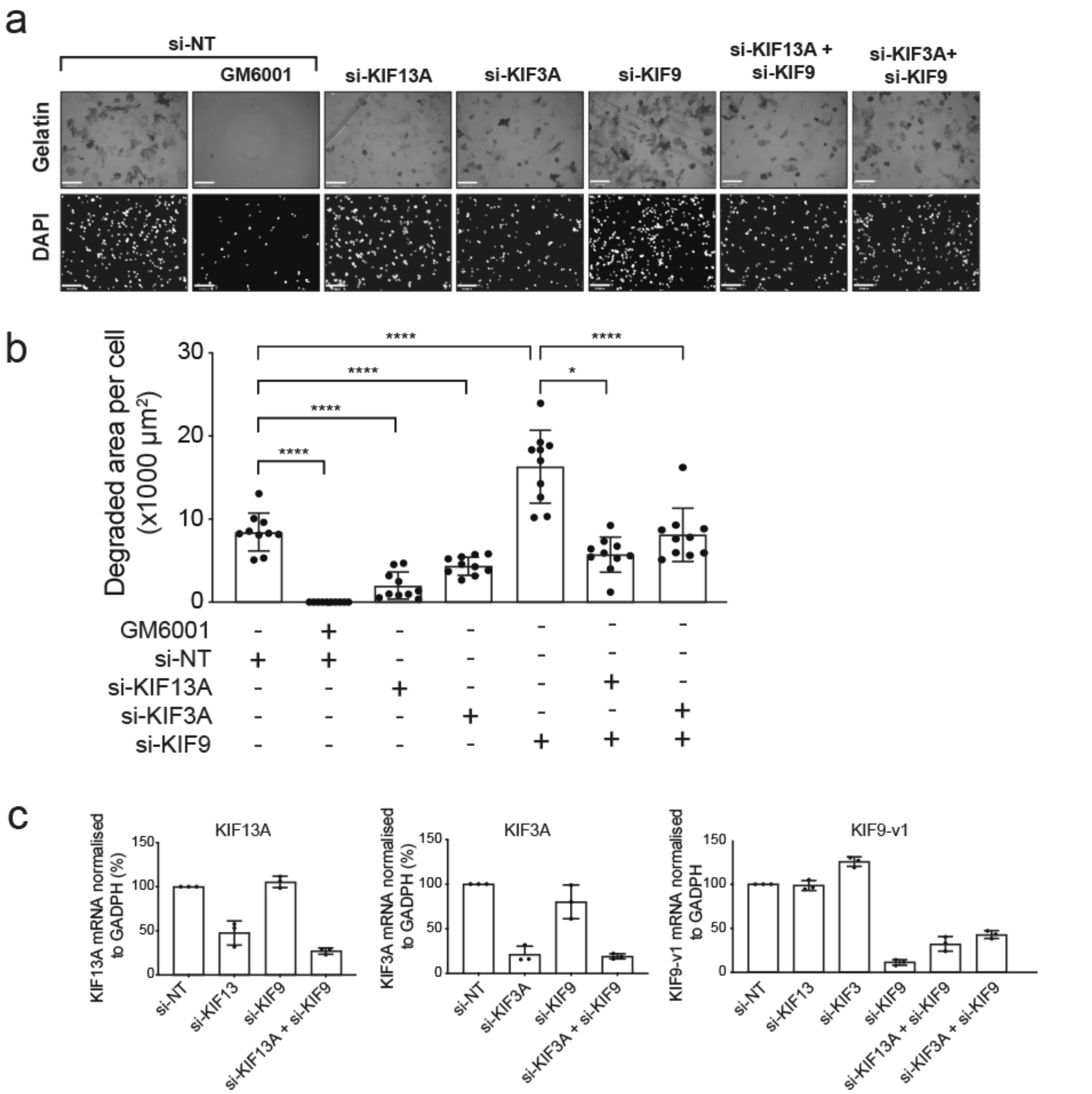

Figure S4. Increased gelatin film degradation upon KIF9 knockdown is due to increased KIF13A and KIF3A-dependent vesicle transport of MT1-MMP

a. HT1080 cells were transfected with siRNA for KIF13A, KIF3A, and KIF9 and subjected to gelatin film degradation assay. Cells were counterstained with DAPI (lower panels). The scale bar is 130µm.

b. Quantification of the degradation area (µm2) per cell. Data are presented as the mean of ten independent microscopic fields of view and are representative of three independent experiments. P values were calculated by the Student T-test. Data are shown as mean ± SD. ****p>0.0001; *p>0.05.

c. Efficiency of KIF knockdown by one-step RT-PCR. Quantification of KIF13A, KIF3A, and KIF9-v1 mRNA fold changes relative to GADPH. The data are representative of three independent experiments.

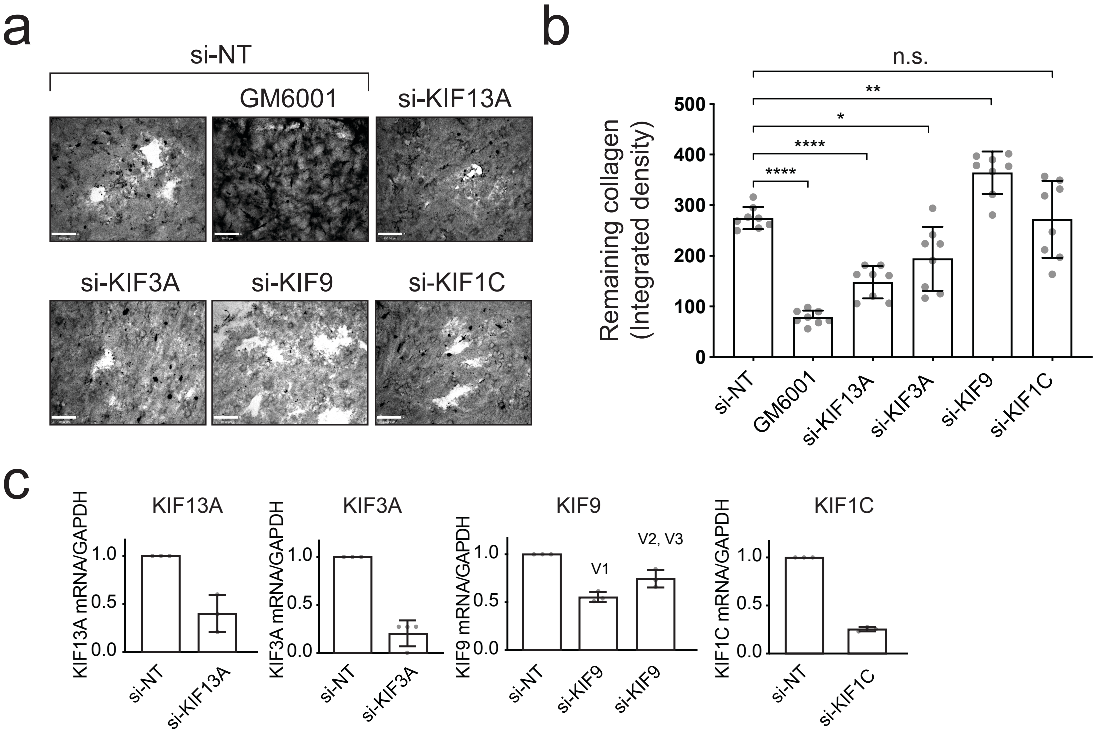

Figure S5. Effect of KIF knockdown on MT1-MMP activity in RASF

a. RASFs were transfected with siRNAs targeting the selected KIFs and subjected to collagen film degradation assay in the presence or absence of GM6001 (10 μM). Scale bars, 130 μm.

b. Quantitative data of the integrated density of the collagen layer in RASFs transfected with the specified siRNAs. Data are presented as the mean of eight independent microscopic fields of view and are representative of five independent experiments. One-way ordinary Anova with Tuckey's multiple comparisons test. Data are shown as mean ± SD. ****$P < 0.0001$, **$P < 0.01$, *$P < 0.05$, n.s., non-significant.

c. Efficiency of KIF knockdown assessed by RT-PCR in RASFs was shown as graphs. The data are representative of three independent experiments.

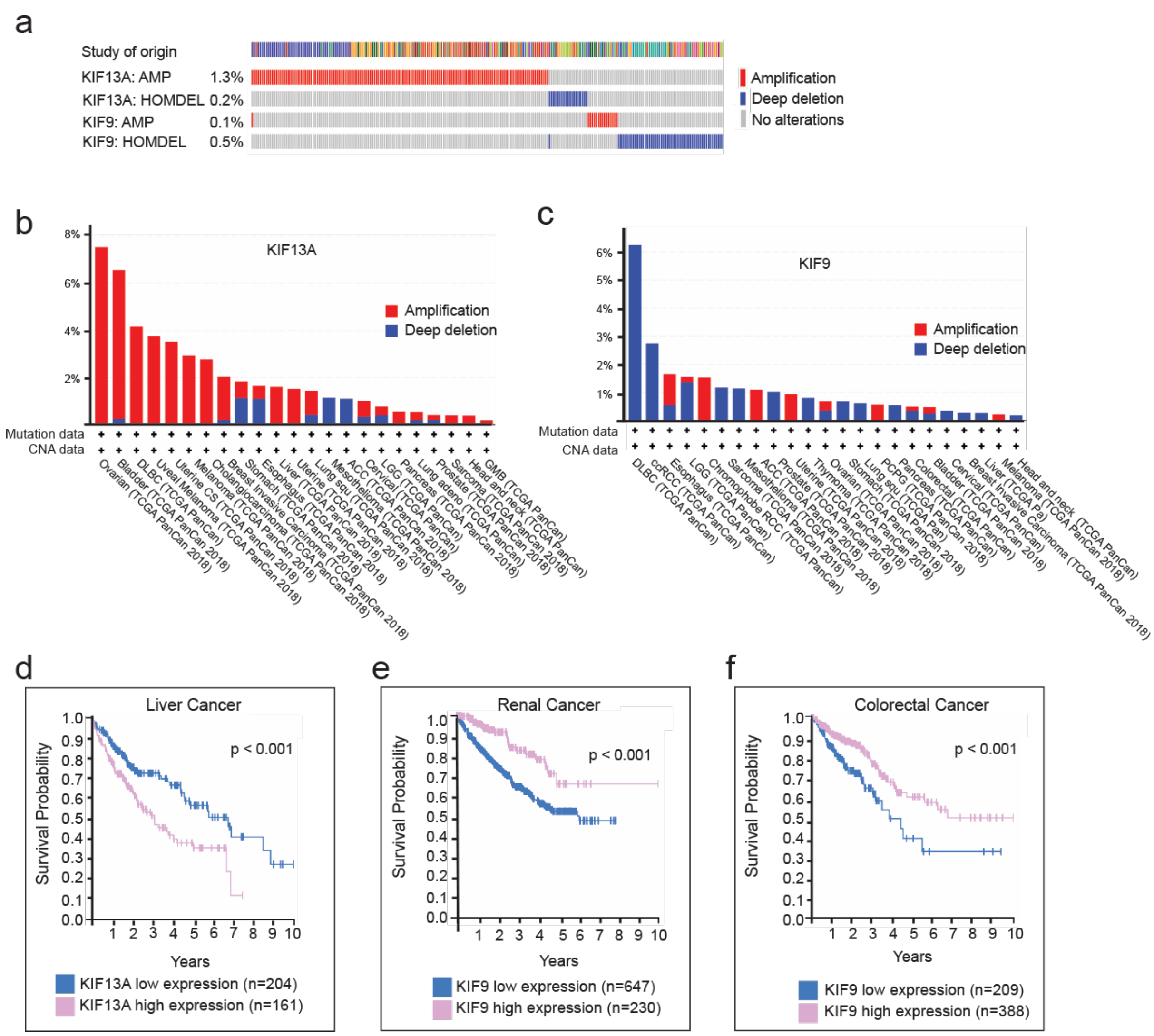

Figure S6. Genomic analysis of KIF13A and KIF9.

a. The oncoprints of KIF13A and KIF9 were identified. Genetic alterations of KIF13A and KIF9. The columns represent patients from studies within TCGA, and the rows gene alterations like amplification and deep deletion.

b. Genetic alterations of KIF13A are summarised according to the study type. Amplifications are shown in red and deep deletions are blue.

c. Genetic alterations of KIF9 are summarised according to the study type. Amplifications are shown in red and deep deletions are blue.

d. Kaplan-Meier survival analysis of liver cancer patients with low or high mRNA expression of KIF13A. P-value was obtained with a log-rank test.

e. Kaplan-Meier survival analysis of renal cancer patients with low or high mRNA expression of KIF9. P-value was obtained with a log-rank test. f. Kaplan-Meier survival analysis of colorectal cancer patients with low or high mRNA expression of KIF9. P-value was obtained with a log-rank test.

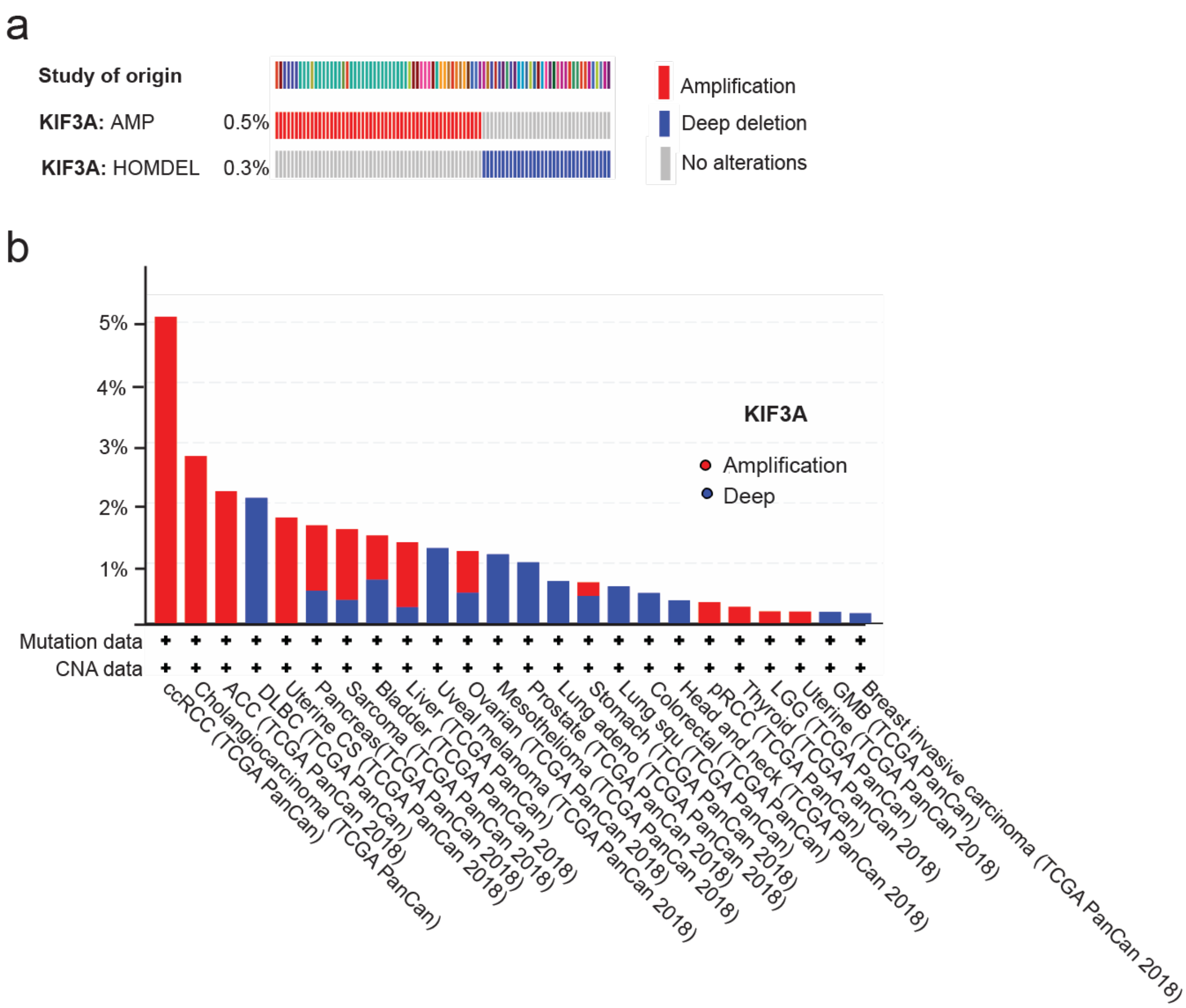

Figure S7. Genomic analysis of KIF3A.

a. The oncoprint of KIF3A was identified. Genetic alterations of KIF3A. The columns represent patients from studies within TCGA and the rows gene alterations like amplification and deep deletions.

b. Genetic alterations of KIF3A summarised according to the study type. Amplifications are shown in red and deep deletions are blue.
